# A High-Fidelity Cell Lineage Tracing Method for Obtaining Systematic Spatiotemporal Gene Expression Patterns in *Caenorhabditis elegans*

**DOI:** 10.1534/g3.113.005918

**Published:** 2013-05-01

**Authors:** Daniel L Mace, Peter Weisdepp, Louis Gevirtzman, Thomas Boyle, Robert H Waterston

**Affiliations:** Department of Genome Sciences, University of Washington, Seattle, Washington 98103

**Keywords:** *C. elegans*, cell fate, gene expression, image analysis, lineage

## Abstract

Advances in microscopy and fluorescent reporters have allowed us to detect the onset of gene expression on a cell-by-cell basis in a systematic fashion. This information, however, is often encoded in large repositories of images, and developing ways to extract this spatiotemporal expression data is a difficult problem that often uses complex domain-specific methods for each individual data set. We present a more unified approach that incorporates general previous information into a hierarchical probabilistic model to extract spatiotemporal gene expression from 4D confocal microscopy images of developing *Caenorhabditis elegans* embryos. This approach reduces the overall error rate of our automated lineage tracing pipeline by 3.8-fold, allowing us to routinely follow the *C. elegans* lineage to later stages of development, where individual neuronal subspecification becomes apparent. Unlike previous methods that often use custom approaches that are organism specific, our method uses generalized linear models and extensions of standard reversible jump Markov chain Monte Carlo methods that can be readily extended to other organisms for a variety of biological inference problems relating to cell fate specification. This modeling approach is flexible and provides tractable avenues for incorporating additional previous information into the model for similar difficult high-fidelity/low error tolerance image analysis problems for systematically applied genomic experiments.

A fundamental interest in developmental biology is to understand the complex cascade of gene interactions that are responsible for allowing an organism to develop from its early stages as an embryo into a fully functional adult. A key component in this understanding lies in knowing when and where genes are being expressed throughout development. Although high-throughput technologies based on cDNAs (*e.g.*, RNA-seq) allow the measurement of expression for many genes across development in a single experiment, they do not allow the practical capture of high-resolution spatiotemporal gene expression data. Recent advancements in fluorescence reporter constructs and microscopy, combined with high-throughput imaging, have allowed the measurement of high-resolution (spatial and temporal) expression data for individual genes across development—resulting in large repositories of image data (Hendriks *et al.* 2006, Hunt-Newbury *et al.* 2007, Tomancak *et al.* 2002). The data encoded in these images complements the data obtained from cDNA expression data sets: where cDNA expression data gives high coverage in the number of genes at the cost of lower temporal and spatial resolution, image data allows for greater-resolution data at the cost of lower coverage in the number of genes.

Akin to other high-throughput technologies, the creation of these image data sets requires multidisciplinary expertise in both biological and computational methods. From a biological perspective, work must be done in the creation, preparation, and imaging for each individual gene. Similarly, significant computational hurdles exist in analyzing the data: the spatiotemporal patterns are encoded in images, and methods must be developed to extract meaningful data (Long *et al.* 2012). These methods are often domain specific and highly customized and have been developed for many model organisms, including *Drosophila* (Kumar *et al.* 2002, Mace *et al.* 2010, Peng *et al.* 2007), zebrafish (Zanella *et al.* 2010), *Arabidopsis* (Mace *et al.* 2006), and *Caenorhabditis elegans* (Bao *et al.* 2006, Santella *et al.* 2010). In this article, we focus on the computational problem of developing high-fidelity methods for tracking individual cell fates across development. Although the output of our method can be used to address a large variety of genomic problems (*e.g.*, natural and phenotypic cell fate variation), we seek to address particularly the problem of obtaining systematic gene expression data across the embryonic development of *C. elegans*.

*C. elegans* is a nematode with a transparent body and a cell lineage that is invariant across wild-type worms (Sulston 1983). By the end of embryonic development the worm produces 671 terminal cells, 113 of which undergo cell death, resulting in a total of 558 cells at hatching for the wild-type hermaphrodite. Upon hatching, some cells undergo another series of divisions before the worm reaches its final adult total of 959 somatic cells. As illustrated in Figure 1, the lineage can be broken down into several well-defined sublineages (often referred to through their founder cells, *e.g.*, AB, C, D), where each individual sublineage yields one or more cell types (*e.g.*, muscle, hypodermal, neuron, intestinal). Although the worm is 700 million years diverged from humans (Vanfleteren *et al.* 1994), many gene regulatory pathways are conserved between *C. elegans* and humans, which, when combined with the aforementioned attributes, makes *C. elegans* an ideal organism for translational research into human diseases and conditions such as Parkinson disease (Lakso *et al.* 2003), Alzheimer disease (Ewald and Li 2010), diabetes (McKay *et al.* 2003), myopathy (Rebolledo *et al.* 2008), and aging (Park *et al.* 2009).

**Figure 1:**
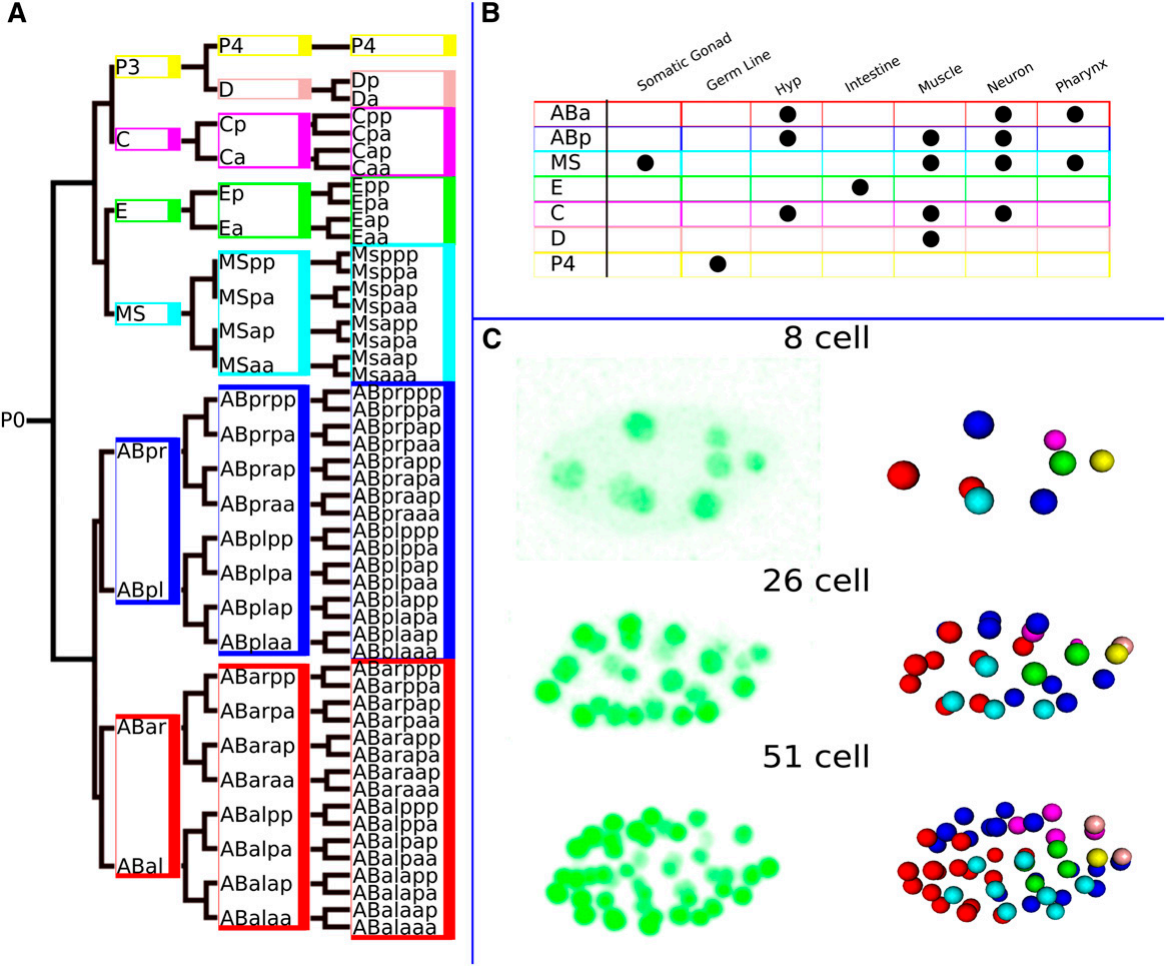
*C. elegans* lineage and tracing: (A) The *C. elegans* lineage follows an invariant division pattern, giving rise to the same 671 terminal leaf cells during embryonic development (early embryonic development to 51 cells shown here). (B) The founder cells are responsible for establishing individual sublineages, which produce one or more primary cell types. (C) Using confocal microscopy, we are able to trace this lineage by using fluorescent promoter constructs to label the individual nuclei from the original images (left). The nuclei are then algorithmically decoded and traced across time (right).

Gene expression in *C. elegans* can be captured *in vivo* by the use of confocal microscopy in conjunction with fluorescent reporter constructs (Murray *et al.* 2008, 2012). In this approach, a ubiquitous histone localized green fluorescent protein construct is used to mark the nuclei, whereas a gene specific promoter driving a red fluorescent protein marks the expression pattern of the endogenous gene. An automated confocal microscopy setup is then used to capture 4D (3D space and time) images across embryonic development. To extract the expression data from these images, we need to develop robust methods to identify and follow the individual cells throughout their development while undergoing a variety of developmental events (*e.g.*, migration, division, death). These methods, often referred to as lineage tracing, provide the full cell identity for each individual cell, allowing the capture of full spatiotemporal gene expression patterns.

Tracing a lineage involves both identifying and linking individual objects (cell nuclei) across time. Lineage tracing shares many similarities to the natural image processing problem of object tracking, which has been intensively studied (Hu *et al.* 2004). Although both problems must confront the issue of identifying and linking objects over time, they each present their own challenges. In the identification process, lineage tracing has to deal with limitations of optics in later stages of development that often result in closely spaced, small nuclei and attenuation effects deeper into the specimen that can lead to difficulty in identifying the individual nuclei. Similarly, in object tracking, in addition to having to deal with irregular backgrounds (*e.g.*, shading), the objects are often 2D projections of an actual 3D scene, which can lead to difficult occlusions. The linking process has several differences as well: in object tracking, objects are often assumed to be independent (or weakly dependent), and have movement characteristics that are modeled using standard physical motion laws. Conversely, lineage tracing deals with objects that follow a stereotypical biological process: cells divide and die off in a very constrained pattern throughout development. These differences in detection and linking behavior limits our ability to use traditional object tracking methods for decoding and extracting data from the images.

Previous methods have been developed for lineage tracing and have been used for decoding high-throughput *C. elegans* image data. An early attempt at lineage tracing, StarryNite, used a separate detection algorithm based on a modified watershed algorithm and a custom distance based algorithm to link the nuclei across time (Bao *et al.* 2006). Improvements were made for detecting the nuclei by using a blob-slice detection algorithm based on the difference of Gaussians (DoG) feature transform of the image with a slightly modified version of the linking algorithm used in StarryNite (Santella *et al.* 2010). Although the blob-slice method decreased the false-positive and false-negative rates, its independent linking and detection algorithms still led to many errors in later stages of development—where the nuclei become closely packed and become difficult to properly ascertain parent/child relationships in divisions/deaths.

We introduce a method for lineage tracing that allows the joint detection and linkage of the nuclei through a hierarchical probabilistic model. This hierarchical framework is able to incorporate additional previous information and constraints in a tractable form. Probabilistic modeling approaches to computer visions problems have become increasingly popular, largely due to the flexibility for incorporating previous information (Ashburner and Friston 2005, Borenstein *et al.* 2004, Levin and Weiss 2006, Tu *et al.* 2005). Much of this work has been done in natural image processing, for whole-scene classification or segmentation problems (Fei-Fei and Perona 2005, Leibe *et al.* 2008). Although enjoying increased attention in the natural image processing community, the biological/medical computer vision communities have been slow to adopt these approaches. Historically, this delay is primarily attributed to the large computational and memory intensive requirements of such models, which does not lend itself well to the already computationally and memory-intensive data sets present in the biological/medical field.

This article distinguishes itself from previous work on lineage tracing in three major aspects. First, we design a hierarchical probabilistic model that allows us to incorporate both a more robust bottom-up detection method with top-down prior information on the biological development. We refer to this model as the Statistically Applied Lineage Tracing model, or SALT. Second, we develop empirical reversible-jump Markov Chain Monte Carlo (RJMCMC) methods that allow us to address detection and linkage simultaneously to decode the *C. elegans* lineage in a more unified fashion. Finally, we validate the model and compare it with previous *C. elegans* lineage methods, demonstrating that the improved detection and reliability have allowed us to extend our lineaging capability to later stages of embryonic development. This increase in resolution and throughput has been paramount for our approach of obtaining systematic genomic spatiotemporal expression data.

## Materials and Methods

**Figure 2:**
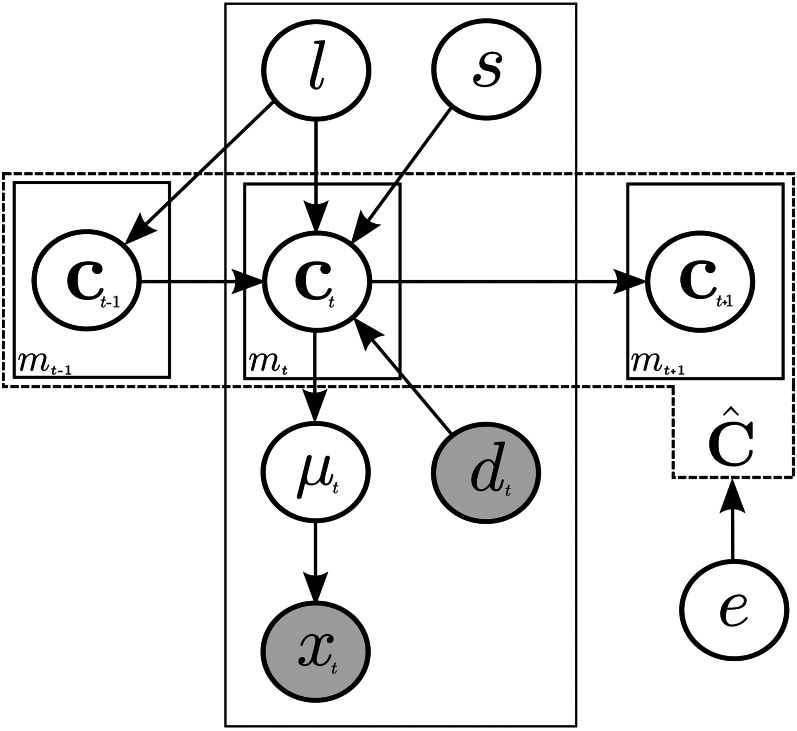
SALT model: a condensed graphical representation of the model. Variables are described using circles, where gray circles represent observed data. Boxes represent multiple occurring values and arrows describe dependencies between variables. In this representation, we describe the lineage of the tree using a variable number of superquadrics *c_j,t_*, where *j* and *t* are indices of the nuclei and time. The superquadrics are linked through time as shown using the arrows, and the aggregation over all the time points forms a tree, represented as ĉ. We represent two types of information in our model, bottom-up information and top-down information. The bottom-up information contains dependencies between the image data and the lineage, and consists of time series of 3D confocal microscopy images, denoted *x_t_*; a feature-transformed image obtained from the original images, denoted *d_t_*; and an induced mean field μ*_t_*. The top-down information consists of various constraints trained on prior lineage data/experiments, and include: measures on spatial distance, size, number, denoted *s*; dependencies describing how cells link through time *l*; and a time to event model on the lifespan of the nuclei, *e*.

Although the *C. elegans* lineage is often displayed in a tree-like fashion (Figure 1A), it is also possible to represent this in a more formal fashion as described here and shown graphically in Figure 2. Let *c_j,t_* be the *j^th^* nucleus at time *t*. We refer to the collection of all *m_t_* nuclei at time *t* as $c_{.,t}$, and the collection of all nuclei across all times as $c_{.,.}$. Because the lineage forms a developmental tree, we can represent this time dependence by establishing parent/children relationships; let *e_p_* (*c_j,t_* ) and *e_c_* (*c_j,t_* ) be the parent set and children set functions for *c_j,t_*. An edge exists between two nuclei if they exist within their respective children and parent sets $E\left(c_{j,t},c_{k,t+1}\right)=1\Leftrightarrow c_{j,t}\varepsilon e_{p}\left(c_{k,t+1}\right)\;\text{and}c_{k,t+1}\varepsilon e_{c}(c_{j,t})$. The number of children define the developmental event (zero children represents a death, one child is a continuation (same cell, new observed nucleus), and two children represents a division with new daughter nuclei). Because divisions and deaths are important developmental events, we further define *N* to be the set of all nuclei that are the result of a division or birth in a tree $c_{j,t}\varepsilon N||e_{c}(e_{p}(c_{k,t}))|=0,2$, and *V* the set of all nuclei that die or divide $c_{j,t}\varepsilon V||e_{c}(c_{k,t})|=0,2$. $N_{i}$ and $V_{i}$ represent the start and end of a branch *B_i_*, where we define a branch to be the collection of all continuous children from *N_i_* to *V_i_*, $B_{i}=\{N_{i},e_{c}(N_{i}),e_{c}(e_{c}(N_{i})),\ldots ,V_{i}\}$. The branch identifier $I_{b}(c_{j,t})$ is used to define the relationship between a nucleus and its containing branch: $c_{j,t}\varepsilon B_{i}\Leftrightarrow I_{b}(c_{j,t})=B_{i}$. Completing this formalization, we set ĉ to be the collection of all nuclei $c_{.,.}$, edges *E*, branches *B*, and development times *o*, *s*, *ĉ* = {*c*_.,._, *E*, *B*, *o*(*e*_[_*_p/c_*_]_), *s_t_*}. *s_t_* is the continuous normalized development time at image capture observation *t*, and *o*(*e*_[_*_p/c_*_]_) is the occurrence time of the parent and children edges respectively (described in the *Methods* section).

Although the previous approaches to lineage tracing had used spheres to represent the nuclei, we use a superquadric representation (Barr 1981). A superquadric is an extension of a sphere that includes additional parameters that allow us to more properly model the anisotropic shape characteristics that are inherent to dividing nuclei. Superquadrics have been used in the computer vision community before, often to describe nonparametric shape models (Terzopoulos and Metaxas 1991). A full superquadric representation would require 10 parameters: three location parameters *x*, *y*, *z*, three axis parameters *a*_1_, *a*_2_, *a*_3_, two exponent parameters *e*_1_, *e*_2_, and two rotation parameters *r*_1_,*r*_2_. We instead reduce the dimension of our superquadric to six superquadric parameters and one additional mean parameter *μ* (described in the *Methods* section): *c* = (*x*, *y*, *z*, *a*_1_, *a*_2_, *r*, *μ*). We assume the first axis and the third axis are linked *a*_3_ = *a*_1_, the second rotation is fixed *r_2_* = 0, and that the minor exponent and minor axis are ratio linked *e*_2_ = *a*_2_*/a*_1_. The first exponent parameter is fit during the training of the model. To account for differences in physical voxel size, all representations of coordinates and calculations are done in physical space. Conversion between the physical coordinate space and image space can be done using fast offset and scaling methods.

With the lineage and shape of the nuclei defined, we can introduce a hierarchical probabilistic model that will include previous information about the development of *C. elegans* for high-accuracy decoding of the lineage. Our hierarchical statistical model is composed of two types of information: bottom-up information (information obtained from the 4D image data sets), and top-down information (previous constraints about the development of the organism). The bottom-up information is composed of two different components: a generative distribution based on the raw observed image and a discriminative distribution based on the DoG feature transform space. The top-down information is composed of three different components: a general component consisting of various topological distributions that constrain nuclei within a time point (major-axis size, spatial interaction, nuclei number, fluorescence intensity); a linkage component to describe the dynamics of nuclei between time points; and a time to event component that describes the occurrence of developmental events (divisions, deaths). Combined, this model allows us to evaluate a proportional probability of any given lineage trace.

Throughout this section, we use simulated annealing methods wrapped around standard Bayesian sampling techniques (in most cases, a standard slice sampling method) (Neal 2003) to train the model. The full model specification, including all previous distributions and the trained parameters, are described in Supporting Information, File S1.

## Bottom-Up Information

The bottom-up component deals with two sources of information: the raw observed image *x*, and a feature transformed DoG image *d*. For the raw observed image we will describe a shared dependent generative model for all nuclei. The feature transformed image will model the individual nuclei independently using a discriminative model. The combination of a generative and discriminative model on two sources of information provides a more reliable and accurate method for nuclei detection: although each individual distribution is prone to its own false-positives and false-negatives, by combining the two approaches we can provide a more accurate method of detection.

### Generative component

The generative component is used to model the relationship between the individual nuclei and the observed image. This relationship is captured by allowing the nuclei to compose a shared dependent mean field $\mu_{.,t}^{\left|g\right|}$ that induces a generative distribution on the observed image. This shared dependency from the mean field is important, as in later stages of development, the compactness of the nuclei exceeds our optical resolution, resulting in the appearance of overlapping and shared fluorescence signal between nuclei.

Intuitively, the generative component describes a relationship between the decoded nuclei and the observed image. During the decoding process, the model will iteratively propose new additional nuclei to the model and evaluate the proposal of the new addition. As part of this process, an expected mean image is generated based on the current and newly proposed nucleus (taking into account information such as the position and intensity of nuclei). The closer the new expected image is to the actual observed image taken with the microscope, the more likely the model is to accept the new configuration.

**Figure 3:**
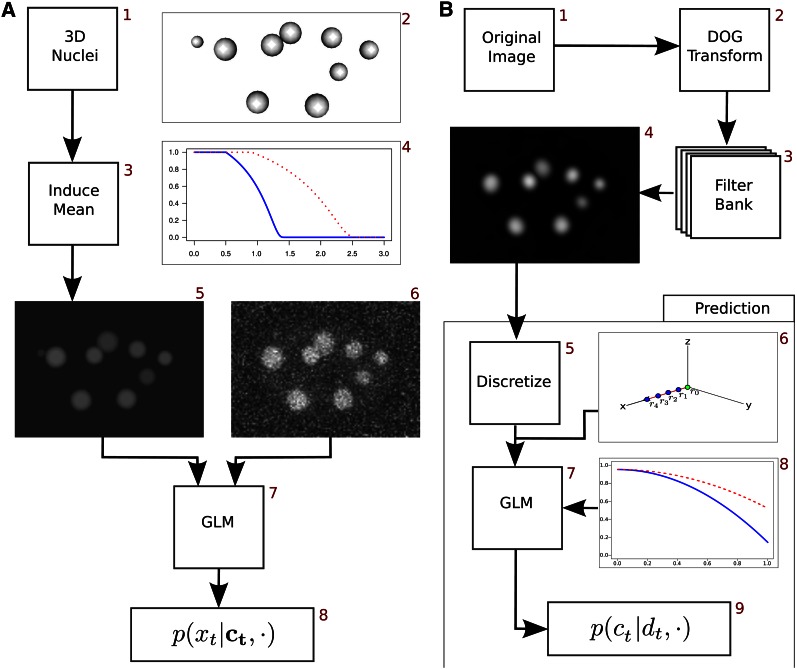
Bottom-up components: (A) The generative component of the model. An expected background model for the observed image is created by using the set of nuclei (2) *c* to induce a mean field by allowing each nucleus to contribute a localized mean to the surrounding voxels. The contribution to each voxel is shown in (4), where the *x*-axis shows the percentage of the mean, and the *y* axis shows the decay based on the normalized distance (distance/radius). The blue line shows the decay in the *x/y* plane, and the red dotted line shows the decay in the *z* plane. The resulting mean field image (5), can be used to form a generative distribution on the observed image *x_t_* (6), by using a generative Laplace distribution (7). (B) The discriminative component of the model. The original image is transformed into a DoG filter bank, an example single element/z-plane of the bank is shown in (4). For each individual nuclei, a discriminative function is created by discretizing the points along equidistant rays extending out from the center of the nuclei (6). A generalized linear model is then fit to model the normalized decay of the DoG image as shown in (8). The *y*-axis is the normalized DoG value, and the *x*-axis is the normalized distance from the center of the nucleus (blue/red lines are the *x/y* plane and *z* plane, respectively). By the use of a series of transformations, a probability distribution (9) can be used to calculate a 0−1 probability on the existence of any point with a specific shape and location configuration.

We define the intensity at location *i* at time *t* of the mean field image to be a linear combination of a background mean, and a weighted value from every individual nucleus. $\mu_{i,t}^{\left|g\right|}=\mu_{b}^{\left|g\right|}+\Sigma_{j}^{m_{t}}\left[f\left(c_{j,t},l\left(\mu_{i,t}^{\left|g\right|}\right)\right)c_{j,t}^{\mu }\right]$, l(⋅) is a location function that returns the x, y, z location of an object (*e.g.*, a voxel location). The summation occurs over the fluorescence mean parameter $c_{j,t}^{\mu }$ for all nuclei within this time point ($m_{t}$), where $\left[f(c_{j,t},l(\mu_{i,t}^{\left|g\right|}))\right]$ is the normalized anisotropic kernel function. This is a piecewise function that overall decreases in value the further the pixel is from the center of the nucleus. The anisotropic nature of the function leads to a slower decrease in the *z* plane than the *x/y* planes to account for attenuation effects that are consistent with confocal microscopy. A graphical representation of this distance function is shown in Figure 3A. 4 and is described in File S1.

The mean field induces a generative distribution on the observed image:

$$p(x|\mu ,b,\cdot )\propto L_{1}(x|\mu^{\left|g\right|},b_{1})L_{2}(\hat{x}|{\hat{\mu }}^{\left|g\right|},b_{2})$$

where *L*_1_ and *L*_2_ and are modified Laplace distributions based on the actual (x,μ) and first derivative$\left(\hat{x},\hat{\mu }\right)$ respectively of the mean and observed values. The combination of both distributions is necessary for both sampling and accuracy reasons: *L*_1_ provides a convex surface that is easy to sample individual nuclei with but is often prone to errors in determining the minor axis and rotation, whereas *L*_2_ provides a nonconvex surface that is difficult to sample from, but is more accurate with determining the minor axis and rotation. The Laplace distributions are normalized to adjust the weight of the generative model compared to all other components in the model: because the area of the superquadric increases at the cube of the major axis, the largest nuclei would have an ≈125-fold increase in voxel contributions to the likelihood—significantly adjusting the relative weight of the generative component to the rest of the model. To adjust for this, additional weighting fields *π*^1^ and *π*^2^ are constructed similar to the mean field construction which addresses this imbalance by reweighting the nuclei such that the largest nuclei would have only ≈3-fold increase in voxel contributions than the smallest nuclei.

### Discriminative component

Although the generative component describes a relationship between all nuclei and the raw observed image, the discriminative component describes an independent relationship between each nucleus and the feature transformed image. The discriminative component is more akin to classical feature detection, as it is often used to describe particular regions that “appear” to be a nucleus. Instead of the traditional “bag of features” approaches that are often used, we use a more formal probabilistic approach for the discriminative model. For any proposed nucleus, a generalized linear model is used to assign a 0−1 probability value on the existence of a nucleus at a given location for any given superquadric parameters. Because the discriminative component is often looking for subtle features of the image space, it is often able to pick out small changes in image intensity that would otherwise be missed. As a result, it is generally more sensitive than the generative model, but less specific.

To fit the discriminative model, we transform the original space into a bank of DoG images (Marr and Hildreth 1980), where *d*_b_ is the *b*^th^ filter bank image, $b\varepsilon H|b=\alpha_{0}+\alpha_{1}r,r=1,2,\ldots ,n$, $\alpha_{0}$ is the starting bank, $\alpha_{1}$ is the spacing of the banks, and *n* is the number of banks. As can be seen in step 4 of Figure 3B, the feature space has peaked values at the center of the nucleus that decrease in value as it extends away from the nucleus. This decay differs from the generative model because it is a strictly decreasing function based on the normalized distance of the superquadric (compared with the piecewise disjoint decreasing function). To model this decay, we define a set of points for nucleus *c_j_*, $R_{c_{j,t}}^{\left|d\right|}$ that are constructed by selecting evenly spaced points along a set of evenly angular spaced rays from the center of nucleus $x\varepsilon R_{c_{j,t}}^{\left|d\right|}|x=Ayw+l(c_{j,t}),w=\{0.25,0.5,0.75,1.0\}$, *y* is a unit vector normalized to the length of the major axis, and *A* is an evenly angled spaced transformation matrix described in File S1 and shown in step 6 in Figure 3B. Because the location of the sampled points do not lie on a standard lattice, a second-order cubic spline interpolation method is used to calculate the values.

Let *r*_i_ be the *i*^th^ element in set $R_{c_{j,t}}^{\left|d\right|}$. We set $\hat{r_{i}}$ to be the DoG value of the discretized point normalized at the filter bank set at the nucleus major axis *a*_1_, by the value at the center of the nucleus *r*_0_: $\hat{r_{i}}=d_{a_{1}}(r_{i})∕d_{a_{1}}(r_{0})$. This decrease of values from the center of the nucleus is modeled using a multivariate normal distribution:

$$\hat{r_{\cdot }}∼N(\mu^{\left|d\right|},\Sigma^{\left|d\right|})$$

An anisotropic angular distance function is used to model the expected value of the discretized values

$$\mu^{\left|d\right|}=1-\beta_{1}^{\left|d\right|}S_{e}{\left(R_{c_{j,t}}^{\left|d\right|}\right)}^{2}$$

where $S_{e}{\left(R_{c_{j,t}}^{\left|d\right|}\right)}^{2}$ is an anisotropic distance function and $\beta_{1}^{\left|d\right|}$ is a parameter that describes the rate at which the DoG value decreases and is show in step 8 of Figure 3B. In later stages of development, this mean level is often distorted by closely spaced neighboring nuclei. To account for this variability, we model a full covariance structure that captures the dependency between closely spaced points:

$$\Sigma_{i,j}^{\left|d\right|}=\sigma^{2,|d|}k_{0}^{\left|d\right|}(i,j)k_{1}^{\left|d\right|}(i,j)+\varepsilon^{\left|d\right|}$$

$\varepsilon^{\left|d\right|}$ is an individual error that is on the diagonal only $\varepsilon_{i,j}^{\left|d\right|}=0,i\neq j$ and $k_{\cdot }^{\left|d\right|}$ are correlation functions described in File S1.

We transform this generative multivariate normal distribution into a discriminative model by converting the probability to log space and using a generalized linear model

$$\begin{array}{ccc} y^{\left|d\right|} & = & \omega_{0}^{\left|d\right|}+\omega_{1}^{\left|d\right|}log(p(\hat{r_{.}}))+\omega_{2}^{\left|d\right|}\tau^{\left|d\right|}(r_{0})\\ p^{\left|d\right|}(c|\cdot ) & = & 1∕(1+exp(-y^{\left|d\right|})) \end{array}$$

$\omega_{\left\{0,1\right\}}^{\left|d\right|}$ are parameters that affect the scaling and offset of the log probability, whereas $\omega_{2}^{\left|d\right|}\tau^{\left|d\right|}(r_{0})$ is a penalty function that ensures that the DoG value at the center of the nucleus is above a certain baseline value. $y^{\left|d\right|}$ is wrapped around a logistic regression model that re-normalizes the value between [0−1], where any value greater than 0.5 would be indicative of the presence of a cell nucleus. As the model normalizes the DoG value to the center baseline pixel, the sensitivity/specificity trade-off comes from $\omega_{2}^{\left|d\right|}\tau^{\left|d\right|}(r_{0})$. Small values would pick up many of the dimly lit cells (*e.g.*, Z2, Z3) but would result in many false positives from image artifacts (general intensity, cover slip). This parameter is learned while training the remainder of the parameters.

## Top-Down Model

Although the bottom-up components deal with information from the observed and transformed images, the top-down components deal with information pertaining to the development of the embryo and is obtained from previously semiautomated methods for tracing the *C. elegans* lineage (Bao *et al.* 2006). The models are trained from a collection of 4D images (large collection of 2D TIFF images) as well as comma delimited files describing the location and parent/child relationships between nuclei. Taken together, we believe in combination with appropriate data input transformations, the method could be readily extended to similar lineage tracing problems in other organisms. A fundamental aspect of the top-down model is a series of components for modeling the relationships between nuclei across time, allowing us to simultaneously predict and link the cell nuclei during the decoding phase. The remaining top-down information consist of previous constraints on either the detection or on relationships between nuclei that allow us to improve the detection and linking accuracy of the model.

Throughout development, many of the topological constraints vary depending on the time of development (*e.g.*, as developmental time progresses, cells become smaller, times between divisions become longer). To more appropriately model this, we use kernel functions to induce time varying parameters as described in File S1. Although this provides more accurate models for the topological constraints, it also introduces an additional complexity during the decoding process as the normalized development time becomes an additional parameter to be sampled. Although the true dynamics of development can be more properly modeled using the cell generation rather than the development time, this approach was avoided as small errors in linking early in development would have a cumulative effect on all remaining daughter nuclei. An intermediate approach of using mixture models to account for heterogeneity in nuclei generation had a similar effect, where decoding the lineage was difficult as daughter cells would often inherit the mistakes of its parent lineage. The coarser approach of using development time and aggregate measures over all nuclei provided a higher and more accurate decoding.

### General top-down components

The general top-down components consist of four independent distributions on various topological constraints: nucleus size, number of nuclei, individual fluorescence, and spatial interaction. The nucleus size consists of two distributions based on the major and minor axis of the nucleus. For the major axis, we use a time varying normal distribution

$$C_{1}^{a}∼N(\mu_{t,1}^{\left|s\right|},\sigma_{t,1}^{2,|s|})$$

**Figure 4:**
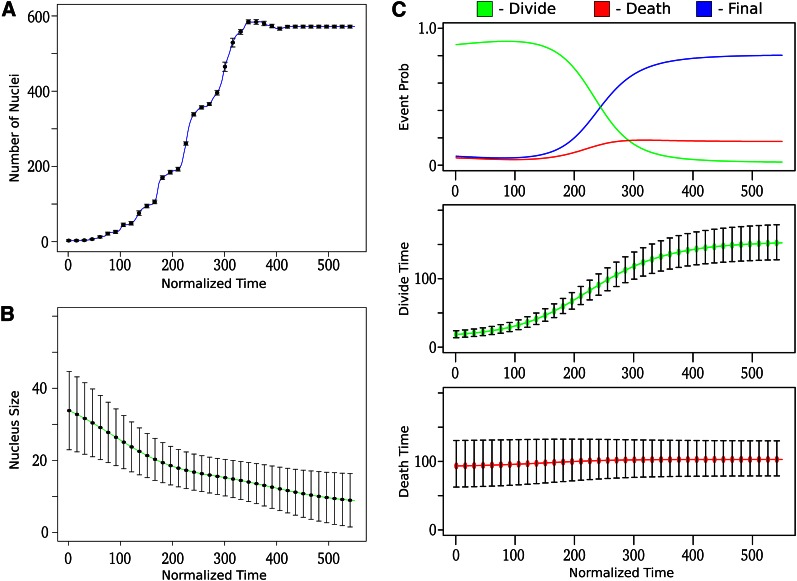
Top-down components: Trained parameters for various top-down components, x-axis refers to the normalized time, mean levels are shown with points, and error bars represent one standard deviation. (A) Number of nuclei as a function of normalized time. (B) Size of the nucleus as a function of normalized time. (C) Time to event model as a function of normalized time: (top) the proportional probabilities of an event to occur; (middle) the timing between divisions; and (bottom) the death event timing.

As can be seen in Figure 4, the size of the major axis becomes smaller as time progresses. For the minor axis, we use a fixed normal distribution

$$log(C_{1}^{a}/C_{2}^{a})∼N(\mu_{2}^{\left|s\right|},\sigma_{2}^{2,|s|})$$

Like the major axis distribution, the distribution on the number of nuclei is also a time varying normal distribution:

$$M_{t}∼N(\mu_{t,3}^{\left|s\right|},\sigma_{t,3}^{2,|s|})$$

The individual fluorescence of each nuclei is defined as follows:

$$C_{.}^{\mu }∼N(\mu_{1}^{\left|g\right|},\sigma_{1}^{2,|g|})$$

The last general top-down distribution is the spatial interaction distribution. To prevent nuclei from directly overlapping, we use a mixed soft/hard core penalization distribution. This distribution is defined as:

$$p(c|\lambda ,\cdot )=exp(-\Sigma_{j,k}h(c_{j},c_{k}))/Z$$

where $h(c_{j,t},c_{k,t})$ is a penalty function from point *c*_i_ to *c*_j_:

$$h(c_{j,t},c_{k,t})=\{\begin{array}{c} \begin{array}{cc} 0 & ifS_{e}(c_{j,t},c_{k,t})>\tau_{0}^{\left|s\right|}\\ \infty & ifS_{e}(c_{j,t},c_{k,t})<\tau_{1}^{\left|s\right|}\\ \lambda^{\left|s\right|}e^{-0.5\frac{\left(S_{e}(c_{j,t},c_{k,t})-\tau_{0}^{\left|s\right|}\right)}{\left(\tau_{1}^{\left|s\right|}-S_{e}(c_{j,t},c_{k,t})\right)}} & \mathrm{otherwise} \end{array} \end{array}$$

This penalty function prevents cells from becoming too close through stepwise constraints: as points enter a soft core distance $\tau_{0}^{\left|s\right|}$, they become slowly penalized based on their proximity to each other until they reach a hard core distance $\tau_{1}^{\left|s\right|}$, where the penalty goes to infinity and the probability of the configuration goes to 0.

The constraints described allow the model to eliminate strong outliers or unexpected nuclei from the decoding. A large 4-μm radius nucleus would be appropriate in early stages of development but would not be expected in later stages of development; rather, the model would be more likely to find two separate nuclei of 2 microns each. Additionally, as the model is often accurately searching for the most optimal solution, the previous constraints prevent the model from searching in parameter spaces that are outside the range expected from the developing embryo, which improves runtime performance.

### Time-dependent top-down component

The previously described components have dealt with the dependence of nuclei within a time point, but not across time points, and can be loosely thought of as the “detection” part of the model, but not the “linking” part, of the model. To establish a linking relationship across time, we model both the dynamics of the cell movement, as well as the timing and fate of the development. For the dynamics, we model the relationship between time points *t* and *t* + 1 using a conditional random field to capture parent child movement constraints (*e.g.*, cell movement, major axis change, division angle). Our linkage component is closely related to the time dependence of a particle filter model, and is merely a modification with additional constraints. Conversely, the timing and fate of the development component deals with the eventual (sometimes unobserved) probability of a developmental event occurring. When a cell arises from the fusion of the pronuclei or from a division, it is “predestined” to undergo one of three developmental fates: it will divide, it will die off, or it will reach its final differentiated cell type. A time-to-event model is used to describe the probability and timing of these events. It is important to note that the scope of each of these components differ. Where the dynamic submodel considers the relationship between nuclei in adjacent time points, the time-to-event submodel considers the aggregate relationship of all cells within a branch across multiple time points.

### Time-to-event component

As described, the time-to-event model deals with the timing and occurrence of developmental events: divisions, deaths, final differentiation. Time-to-event models are often used in clinical studies for survival analysis of patients with disease (Hosmer *et al.* 2008). This component can be abstractly thought of as a distribution on the development lineage tree itself. Placing restrictions on the tree allows the model to prevent unexpected occurrences (*e.g.*, early cell deaths, fast divisions, missed divisions). These constraints are crucial in later stages of development, where the close proximity and attenuation effects make cell divisions and cell deaths difficult to properly follow individual nuclei across time.

A crucial requirement of the time to event model is the ability to account for the changing characteristics of the developing embryo. Early development is characterized by rapid divisions that quickly establish the main founder cells responsible for the individual sublineages. With the exception of the germline cells Z2 and Z3, none of these cells have entered their final differentiated state, and there are no deaths. Later in development, the organism enters organogensis, and the time between divisions increases. Cell deaths are common in this stage, and many cells are nearing their final embryonic cell state. We model this using a time varying time to event model.

Let $S(B_{i})$ be the indicator function of the ending development event for branch $B_{i}$, where $S(B_{i})=\{0=\mathrm{division},1=\mathrm{death},2=\mathrm{final}\}$. We describe the probability of this branch as

$$p(B_{i}|e,\cdot )=\pi_{S(B_{i}),t}^{\left|e\right|}p_{S(B_{i})}^{\left|e\right|}(d(B_{i}))$$

where $\pi_{S(B_{i})}^{\left|e\right|}$ is the base probability of the event, and $p_{S(B_{i})}^{\left|e\right|}(d(B_{i}))$ is the probability that a branch exists with a duration length of $d(B_{i})=\sqrt{o(e_{c}(V))-o(e_{p}(N))}$. We introduce o(…) as the developmental timing of the start or end of the branch. These are additional parameters of the model and are sampled during the decoding phase, bound by their respective normalized times. The base probability of an event at time *t* is:

$$\pi_{i,t}^{\left|e\right|}=\frac{exp\{\rho_{i,t}\}}{\sum_{j}^{3}exp\{\rho_{j,t}\}}$$

The duration between events is modeled using a time varying Gaussian distribution based on the square root of the branch duration:

$$p_{S\left(B_{i}\right)}^{\left|e\right|}\left(B_{i}|\cdot \right)\propto exp\left(-0.5^{*}{\left(d\left(B_{i}\right)-\mu_{S\left(B_{i}\right),t}^{\left|e\right|}\right)}^{2}/\sigma_{S\left(B_{i}\right),t}^{2,|e|}\right)$$

where $\mu_{S(B_{i}),t}^{\left|e\right|},\sigma_{S(B_{i}),t}^{2,|e|}$ are the mean and variance for the event. The timing of what constitutes a cell death often varies based on the literature, and can mean many things. In our representation, the timing of cell death is the time from when a cell divides until it essentially disappears and becomes “untrackable.” Because this timing is directly obtained from previous lineage experiments, we expect it to vary from other definitions of cell death. The time-varying parameters of the trained model are shown in Figure 4C.

### Linkage component

The linkage component describes the dynamics of the relationships of nuclei between neighboring time points *t* and *t* + 1. For the dynamics, two possible configurations exist between parent and children: cells continue on (one child), or cells divide (two children). The third possible configuration, cell death, is not modeled as there is no cell at time *t* + 1 to model the dynamics; however, the occurrence of timing and cell death is accounted for in the time to event component.

The linkage component describes the fundamental element responsible for establishing relationships between nuclei across two time points. In other models, this relationship is often established through custom heuristics based on the Euclidean distance between nuclei across time, or in the case of particle filters, previous constraints on the dynamics of the cells movement. As we describe, our method extends upon these approaches by developing models that describe specific constraints on how the nuclei change over time (*e.g.*, size, location), and how they respond during division events (*e.g.*, angle of division, division distance).

The dynamics between a parent and its children varies based on whether a nucleus is continuing on or dividing. We model individual state specific dynamics by imposing time varying constraints on the parent child relationships between nuclei. In the single child case (continuation), constraints are placed on the movement of the nucleus (expected to be small), and the change in nuclear size (also expected to be small). For divisions, the case is more complex: the cell often undergoes a stereotypical pattern of division in which the dynamics of each daughter cell are dependent. To account for this dependency, we use five features to model nuclear divisions: the movement of each daughter nucleus (2), the change between the radius of the parent and each child (2), the difference of the change between the radius of the parent and each child, the difference in the movement of both daughter nuclei, and the angle of the division. We use a conditional random field (CRF) to link these constraints (Lafferty *et al.* 2001). The CRF provides a natural way of describing links by incorporating each individual constraint into a feature, and then fitting a feature weight:

$$\begin{array}{ccc} p\left(c_{t}|c_{t-1},\cdot \right) & = & exp\left(\psi \left(x\right)\right)\\ \psi \left(x\right) & = & \Sigma_{k}^{n}\left(g_{k}^{\left|l\right|}\beta_{k}^{\left|l\right|}h_{k}^{\left|l\right|}\left(\cdot \right)\right) \end{array}$$

*ψ* is the summation over all possible feature functions $h_{k}^{\left|l\right|}$, whereas $\beta_{k}^{\left|l\right|}$ are the individual weights for the features. $g_{k}^{\left|l\right|}$ is an interaction term that accounts for some of the covariability of the division features. For the features we use time varying squared error functions: $h_{k}^{\left|l\right|}=-{\left(x_{k}^{\left|l\right|}-\mu_{k}^{\left|l\right|}\right)}^{2}∕\sigma_{k}^{2,|l|}$, where $x_{k}^{\left|l\right|}$ is an observed feature. Because the feature functions are unnormalized log normal probabilities, and the CRF is an exponentiated summation over these functions, the CRF essentially becomes a weighted unnormalized product of Gaussian distributions. We make extensions to the mean and variance to allow it to become more generalized:

$$\begin{array}{ccc} \mu_{k}^{\left|l\right|} & = & \lambda_{k}^{|l|,0}+\lambda_{k,t}^{|l|,1}+\lambda_{k,t}^{|l|,2}e_{t,t-1}\\ \sigma_{k}^{2,|l|} & = & exp\{\gamma_{k}^{|l|,0}+\gamma_{k,t}^{|l|,1}+\gamma_{k,t}^{|l|,2}e_{t,t-1}\} \end{array}$$

This extends previous time varying parameter specifications to include an additional parameter $e_{t,t-1}$, which is the elapsed development time for the link: $e_{t,t-1}=o(c_{j,t})-o(e_{p}(c_{j,t}))$. By allowing the model to change based on the duration between time points we provide a dynamic relationship for the model: as the spacing between time points increases, the mean and variance of the feature space changes (*e.g.*, the variance on the cell movement increases while the mean division angle decreases). This extension allows us to decode images taken from varying time spacings of image capture. Time varying parameter representations are shown in Figure S1.

## Decoding the Lineage

The hierarchical probabilistic representation allows us to evaluate the probability of any given lineage as the proportional product of the generative, discriminative, general top-down, time to event, and linkage submodels:

$$p(\hat{c})\propto p(x|\hat{c},g,\cdot )p(\hat{c}|d,\cdot )p(\hat{c}|s,\cdot )p(\hat{c}|l,\cdot )p(\hat{c}|e,\cdot )$$

This proportional product can be used to determine the most probable configuration for any given 4D image series by employing hybrid simulated annealing and RJMCMC to decode the lineage. RJMCMC is an extension to standard MCMC methods that are often used *in trans*-dimensional model selection problems such as variable selection, mixture models and factor analysis (Green 1995). In our particular case, the number of nuclei and their link through time is an unknown. As a result, determining the most probable lineage becomes a *trans*-dimensional model selection problem. Similar work using RJMCMC methods have been applied in computer vision for identifying cells (Al-Awadhi *et al.* 2011), detecting trees (Perrin *et al.* 2006), plant branching (Schlecht *et al.* 2007), road extraction (Lacoste *et al.* 2010), and human tracking (Zhao *et al.* 2008). Although particle filters can also be used to address the uncertainty in nuclei in the lineage, they are known to have difficulties in high dimensional problems (558 cells at the end of embryonic development), where the number of particles needed to accurately represent the underlying model is both computationally and memory prohibitive (Peursum *et al.* 2010, Snyder *et al.* 2008).

We decompose the RJMCMC methods into two distinct set of moves: single time point RJMCMC moves, and multi-time point RJMCMC moves. The single time point RJMCMC moves behave similar to traditional decoding approaches such as particle filters, by addressing each individual time point in a sequential fashion *t* = 1, 2, 3,… through a series of reversible jumps moves such as adding a point, or removing a point within a given time point. The multi time RJMCMC methods are akin to back filtering methods of particle filters in that they can occur over multiple time points and at any current or previous time and are generally defined by moves that affect the tree of the lineage, such as: adding a branch to the lineage, or removing a branch from the lineage. By combining both of these approaches, we are able to address issues that are difficult to account for using standard sequential RJMCMC methods, such as false deaths or lack of detecting a daughter cell during a division. The full algorithmic details on the selection of moves, updates, and all details on empirical proposals are described in File S1.

### Single-time RJMCMC

The single-time steps deal with moves on a single individual time point. These consist of six moves, four RJMCMC moves, and two standard MCMC moves. The four RJMCMC moves are: add a point, remove a point, split a point into two points, and merge two points into one point. Adding a point *k* = *m*_t_ + 1 to time point *t* consists of a transition from $\hat{c}\to {\hat{c}}^{’}$, where ${\hat{c}}^{’}=\hat{c}\cup^{\;}c_{k,t}$. To simulate an accurate draw from the posterior of ${\hat{c}}^{’}$, we draw a new set of parameters for $c_{k,t}$, where the shape parameters are drawn from their time varying prior distributions (uniform if no previous exists (rotation)), and the location is drawn from an empirical proposal distribution. Standard MCMC methods are used to update the parameters of the new point using a slice sampler (Neal 2003) and are intermixed with a linking step to establish parent/child relationships from the previous time point t−1 as described in the following linking step. After updating the parameters and linking the parents for the point, the addition of the new point is then accepted or rejected using the acceptance ratio described in File S1. The remove move performs the opposite, drawing a point *j* from time *t* from an empirical proposal and choosing either to accept or reject the move. The merge move operates by taking two individual points, *c_j,t_* and *c_k,t_*, and merging them into one new point, *c_l,t_*, whose parameters are set to the mean parameters of the previous two points. The merged point then undergoes an updating and linking step similar to the add move and is either accepted or rejected. The split move is the reverse of the merge move, and splits one point *c_l,t_* into two points, *c_j,t_* and *c_k,t_*, where the new parameters are then drawn from the previous distributions, and the location is drawn from an orthogonal split along the location of previous points. The parameters and parents of each new point are then sampled similarly and either accepted or rejected. In addition to the reversible jump moves, there are also two standard MCMC moves. The first is an update point move, which involves updating the parameters and parents of each individual point. The second move, is a relink area move, which uniformly samples a point $c_{j,t}$ from all points at time *t*, selects all points within a radius *r* of *c_j,t_*, and updates their parent cell relationship.

### Multi-time RJMCMC

The multi-time steps are a combination of individual single time add and remove steps that span over multiple time points. These consists of five moves, four RJMCMC moves, and one standard MCMC move. The four RJMCMC moves are: add a branch, remove a branch, adjust start of branch, and adjust end of branch. For the add a branch move, a time point is empirically sampled and proposed to extend backward a length of $k_{1}∼1+\mathrm{Poisson}(\lambda_{1})$ in time using standard MCMC methods to update the parameters for each individual time point. A new configuration is then selected by empirically choosing a branch length from all possible branches ${\hat{c}}_{1}^{\prime },\ldots ,\hat{c_{k}}\prime$ based on their individual probabilities. To maintain balance with the deletion move, the selected branch then undergoes a relinking step, where the end of the new branch is adjusted as described below. The new adjusted branch $\hat{c}\prime$ is then accepted or rejected based on the standard acceptance ratio. The remove branch proceeds in the opposite fashion: a uniformly selected branch $B_{i}$ is selected from the lineage, and undergoes a relinking of the right side of the branch. All points within this branch are then removed from the lineage $\forall c\varepsilon B_{i}$. The new configuration, $\hat{c\prime }$ then undergoes a similar acceptance ratio.

The adjust start and end branch moves involve extending or contracting the start or end of a particular branch of the lineage tree. Let $B_{i}$ be a randomly selected branch of the lineage. The start relinking of $B_{i}$ involves proposing to add or remove a length of $k_{2}∼1+\mathrm{Poisson}(\lambda_{1})$ elements of the branch. The move is considered in both directions for both relinking steps, which results in $2^{*}k_{2}+1$ possible moves (the relinking of the left, right, or the current state). The new state of the model is then selected from the empirical probabilities of all possible moves.

### Parent/child linking step

The parent child relinking step is a sampling step for linking parent/child relationships. For a cell $c_{j,t}$, we propose to link it to a parent cell at time point t−1. Let $Q_{j,t}$ be the set of all points at time t−1 that are within a radius $r∼N(\mu_{d}^{\left|l\right|},\sigma_{d}^{2,|l|})$ of point $c_{j,t}$, where the mean and variance are the expected mean and variance from the distance feature of the division link CRF. For each point in the proposed set, $c_{k,t-1}\varepsilon Q_{j,t}$, with configuration ${\hat{c}}^{k}$, we let

$$r_{j,k}=\frac{p\left({\hat{c}}^{k}|\cdot \right)}{\Sigma_{m}^{\left|Q_{i,t}\right|}p({\hat{c}}^{m}|\cdot )}$$

be the normalized proposed probability of a new parent. A parent is empirically selected from these normalized probabilities. Additional empirical moves are also used to allow for switching moves and for relinking past/future events, and are discussed in File S1.

### General parameters

In addition to the parameters for the nuclei and their links, we sample the normalized development time for each time point in the series. Let *s_t_* be the observed normalized time for time point *t*. A linear model is then fit to *s_t_*: *s_t_* = β_0_ + β_1_*t*, where β_0_ is the starting normalized offset time, and β_1_ is the spacing between time points. The parameters are fit using a weighted posterior that is proportional to the prior cell number distribution of the initially seeded data described in File S1 as well as the cell number distribution of the decoded data and the generative time varying components of the full model (time to event, major axis, and cell numbers). These parameters are updated throughout the decoding of the model.

### Replica exchange model

To improve performance and increase convergence, a local replica exchange model is used (Cheng *et al.* 2005). Throughout the decoding process, a series of *l* possible chains are run, where each individual chain has separate parameters ${\hat{c}}^{q},q=\{1,\ldots ,l\}$, with their own temperature $a_{q}=T^{k}$. For each decoding move (single and multi time), standard updates are mixed with replica exchange moves, where the temperature of each individual chain are proposed to exchange. Let *q*, *r* be two individual replicas, with individual temperatures $a^{q}=T_{m}$, and $a^{r}=T_{n}$. A proposal is made to swap the temperatures of particles q,r with acceptance probability

$$\alpha \left(a_{q}=T_{m}\to T_{n},a_{r}=T_{n}\to T_{m}\right)=\frac{p{\left({\hat{c}}^{q}\right)}^{T_{n}}p{\left({\hat{c}}^{r}\right)}^{T_{m}}}{p{\left({\hat{c}}^{q}\right)}^{T_{m}}p{\left({\hat{c}}^{r}\right)}^{T_{n}}}$$

The acceptance or rejection of each decoding move is performed on the lowest temperature replica:

$$\alpha \left({\hat{c}}^{i\left(T^{k}\right)},{\hat{c}}^{i(T^{k})\prime }\right)$$

where $i(T^{k})$ is the replica indicator of the lowest temperature element (the *k^th^* temperature). After the acceptance or rejection, the individual replicas are all synchronized to the lowest temperature replica: ${\hat{c}}^{i}={\hat{c}}^{i(T^{k})},i=\{1,\ldots ,l\}$. The local replica exchange model allows us to more thoroughly explore the configuration space of the lineage without getting stuck in local optima.

### Postanalysis curation and expression determination

After the image series has been processed as described, a final manual curation step takes place where individual errors are corrected as described in Boyle *et al.* (2006). As part of this curation step, automated methods are run that provide the final cell lineage name using a rule-based system and quantification of the expression across the whole lineage.

## Results

We have developed a method to identify nuclei and trace the cell lineage of the developing *C. elegans* embryo from 4D (3D space and time) confocal microscopy images. Our method differs from previous cell lineage tracing approaches by incorporating prior constraints about *C. elegans* embryonic development into the tracing problem. These constraints are designed to correct many of the errors prevalent in earlier approaches by more explicitly taking into account the natural patterns inherit to development (*e.g.*, expected angle of division, timing between divisions). We modeled these constraints using common statistical approaches and use general aggregate measures across the population of cells to provide a more extensible model. In addition, we also integrate the often two-step approaches for cell lineage tracing (identifying nuclei in a first pass, and then linking nuclei through time in a second pass) into a single unified method. This unified approach, when combined with the previous constraints, allows us to correct for many of the errors that were common with previous methods in later stages of development.

### Validation and accuracy

To assess the performance of this unified approach, we compared our model (SALT) to the two previous full embryonic methods for *C. elegans* lineage tracing of StarryNite referred to as WaterShed-StarryNite (WS-SN) (Bao *et al.* 2006), and BlobSlice-StarryNite (BS-SN) (Santella *et al.* 2010). Although other methods have also attempted to address this lineage problem (Carranza *et al.* 2011, Giurumescu *et al.* 2012, Kang *et al.* 2012) all have error rates on par with the original WS-SN, with none approaching the BS-SN method. Additionally, none of these methods have been evaluated beyond early/mid embryonic development. As we demonstrate, our method is a significant improvement over both the WS-SN and BS-SN methods at both early and later stages of embryonic development.

**Figure 5:**
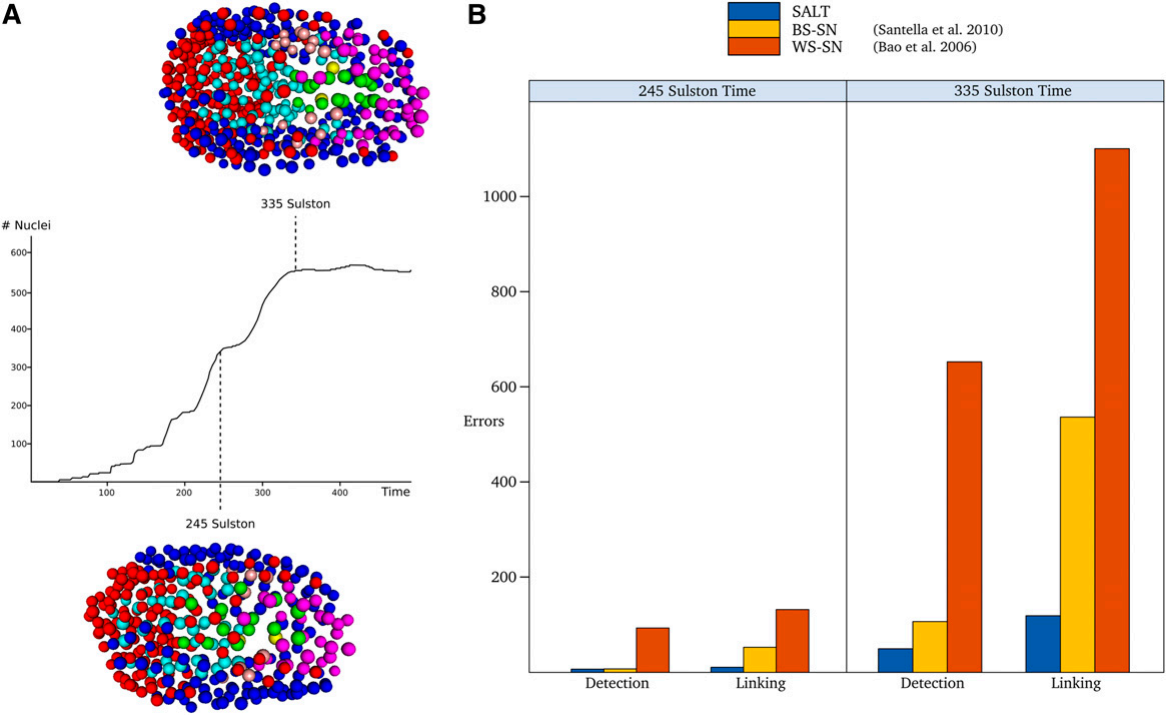
Error rate evaluation. (A) The evaluation was performed at the 245 Sulston time (350 visible nuclei) and 335 Sulston time (550 visible nuclei). 3D representation of the nuclei are shown, colors are identical to colors used in Figure 1. (B) Comparison of three methods: our method (SALT), Blob Slice-StarryNite (BS-SN), and WaterShed-StarryNite (WS-SN). The method was evaluated for two types of errors, detection and linking. Lower bars represent fewer errors and greater accuracy.

We established a quantifiable measure for the accuracy of each method by creating a test set of 12 image series evaluated at two different time stages: the 350-cell stage (245 normalized time, right after the 8th, but before the 9th AB lineage division), and the 550 (550 total visible cells, some of which are undergoing cell death)-cell stage (335 normalized time, right after the 9th, but before the 10th AB lineage division). The 550-cell stage was chosen as an easily identifiable embryonic development time needed to address the varying biological experiment ranges of the individual image series. The error rate for each individual series was assessed on two types of errors: detection errors (expected cells that are missing, or new unexpected cells) and linking errors (*e.g.*, incorrect assignment of daughter cells during division, change of naming between time points). To provide a standardized method for evaluation, each individual method is automatically compared to fully curated data sets as described in detail in Supplemental Methods. Full results of all three methods are shown in Figure 5 and a detailed breakdown and analysis on the types of linking errors are described in File S1.

Our approach outperformed the previous methods in both detection and linking errors. In earlier stages of development (350 cells), both SALT and BS-SN performed similarly in detection abilities, with a near-identical number of detection errors (6.8 *vs.* 7.3). However, in linking the nuclei, SALT performed better, having a 4.9-fold reduction in errors (10.8 *vs.* 52.8). In later stages of development (550 cells), both the detection and linking of SALT were significantly improved compared with the other methods. Our approach had a 2.2-fold reduction in detection errors (49.6 *vs.* 106.8), as well as a 4.5-fold reduction in linking errors (119.0 *vs.* 536.7) for an overall 3.8-fold reduction compared with BS-SN.

The error rate is an important measure of quality because these errors must be corrected through manual curation to obtain an accurate lineage tracing and hence expression pattern. This step is critical since even a single error early in development can cause a cascade effect throughout the remainder of the lineage. A decrease in the number of errors translates into a decrease in editing time for a given developmental stage and also allows us to curate our data sets to later stages of embryonic development. Our evaluation of the editing effort in this paper is based on the number of curation steps required to correct mis-specifications in the lineage. The previously reported edits from WS-SN at the 350-cell stage required a combined 225 curation steps to establish the correct lineage. By contrast, our method required 18 curations at the 350 cell stage, and only 168 curations at the 550-cell stage. Thus, our method effectively performs better at the 550-cell stage than WS-SN at the 350-cell stage, requiring 25% fewer corrections. This significant improvement allows us to follow spatiotemporal gene expression data out to later stages of development than previously possible.

### Detecting expression in final differentiated cells

Extending our lineage capabilities to the 550-cell (335 time) point allows us to address biological questions that would not be possible with earlier approaches. At the 350-cell (245 time) point, many of the cells still require one or more divisions before they reach their terminal embryonic cell fate. The majority of this final differentiation occurs in the AB lineage, where the last remaining division often signifies the specialization of a cell into one of many different subclasses of neurons. Proper specialization of the neuronal sublineage is an intricate process and involves many different genes acting in a complex cascade of gene transcription (Hobert *et al.* 2010). With the extension of lineaging to the 550-cell point, nearly all the individual cells have reached their final state of differentiation in embryonic development.

**Figure 6:**
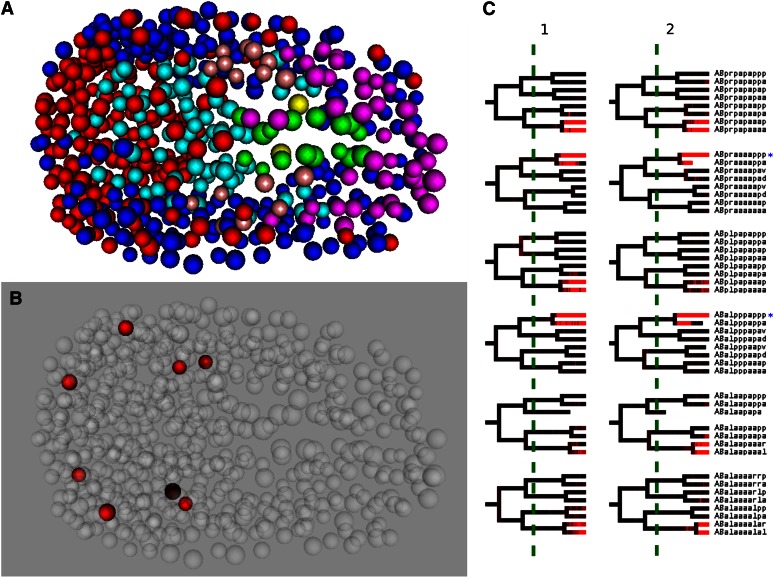
*ttx-3* Evaluation. *ttx-3* Expression: (A) 3D nuclei representation at 335 Sulston time. Individual nucleus color is identical to colors used in Figure 1. (B) Representation with expressing no death cells highlighted. Colors represent intensity. (C) Comparison of two replicates (1 and 2) of ttx-3. Green line represents previous limitations of gene expression (245 Sulston). Blue asterisks represent an individual cell that still has one division cycle remaining.

To illustrate the importance of this increase in fidelity, we show the expression profile of an individual gene, *ttx-3*, at both the previous limit of 350 cells as well as at 550 cells obtained with our new method. *ttx-3* is a transcription factor required for proper functioning of the thermosensory and olifactory response, and is responsible for cell fate specification for a subset of the neurons (Bertrand and Hobert 2009). As can be seen in Figure 6, data at 350 cells fails to capture any of the mid/late onset expression for *ttx-3*. In contrast, our method is capable of detecting the onset of expression that occurs in 12 cells near or after the final cell fate decision. These 12 cells are composed of the six previously detected cells: AINL, AINR, AIYL, AIYR, SMDDL, SMDDR (Bertrand and Hobert 2009), as well as the ASKR and ASKL cells and four cell deaths. As can be seen in Figure 6, ASKL and ASKR appear in both technical replicates and we expect this may relate to differences in detection sensitivity, differences in constructs, or possibly differences in integration sites.

To provide an estimate of how many genes are differentially expressed in late stages of development that our method would be able to detect, we looked at the next closest representation of spatiotemporal data in *C. elegans*: tissue-specific expression data obtained from fluorescence-activated cell-sorted data (Spencer *et al.* 2011). Using a precompiled list of genes with significant expression, we filtered for genes that have no expression in early embryonic expression, but have differential expression in later embryonic stages. Although this filtering provides a stringent criteria for detecting differential late gene expression, we were still left with 4456 potential genes that are candidates for late-onset differential expression. This list, encompassing nearly 25% of the genome, illustrates the importance of having high-fidelity robust methods for our systematic approach to obtain genomic level expression data throughout embryonic development.

## Discussion

By incorporating general prior constraints into the model and simultaneously addressing detection and linking, we have developed a robust method that has significantly reduced the error rate for lineage tracing applied to 4D confocal microscopy images. Our model is more accurate than previous approaches, with an overall reduction in errors of 10.4-fold and 3.8-fold compared with the WS-SN and BS-SN versions. This increase in accuracy has allowed us to follow spatiotemporal gene expression to later stages of development, providing greater resolution data for differentially expressed genes.

Although our method has increased performance in both detection and linking at later stages of development, we expect the increased performance in detection is not the result of an improved detector, but rather from the combined integration of prior data with a unified detection and linking method. Many of the detection errors in later stages can be corrected by accounting for prior constraints and dependency between time: false-positive results are prevented by the general constraints on division appearances and timings, and false negatives are prevented from expectations on deaths. The importance of this integration becomes even more apparent when dealing with assigning relationships to nuclei between time points further on in development. In these later time stages, the nuclei are small and tightly spaced, creating irregular division patterns that often cannot be explained by simple proximity relationships. Many of these issues are often corrected at later sampling times, when constraints on division timing and the presence of new information allows the model to correct previous mis-assignments.

Full 4D imaging of developmental organisms has become increasingly important, and methods to trace the lineage are paramount for biological discovery. Although our current application of cell lineage tracing has been for the direct purposes of obtaining high-resolution systematic gene expression data, robust methods for following cell identity are applicable to a variety of problems and are critical for understanding phenotypic variation through detailed quantifiable measures (cell movement, cell−cell interaction, cell cycle). The benefits of high-fidelity lineage tracing become more apparent when considering organisms or processes with variant cell fate, such as cancer progression, where these quantifiable measures would provide us with a more fine grained analysis of the underlying mechanisms that is not possible with other high throughput methods.

The methods used in our model are generalized linear models that are extensions of standard hierarchical statistical models, with slight modifications to the RJMCMC to allow for empirical Bayesian proposal distributions. With modifications to account for increased variance from more complicated lineages, as well as performance modifications to account for increased number of nuclei (discussed below), all of the bottom-up and top-down distributions used in this model have a direct application to other model organism. Additionally, we expect the more formal probabilistic structure to be amendable to incorporating additional previous information on the development of the organism (*e.g.*, joint lineage tracing of the nuclei with segmentation of the cell wall/membrane), expanding our ability to address new biological problems.

We note that although our method outperformed both WS-SN and BS-SN in accuracy, it does have a drawback in its runtime performance. The total computational and runtime demands of our model are ≈22 hours and 12 GB of ram for the 550 cell (335 time) stage. This is in contrast to WS-SN’s requirement of 30 minutes and 200 MB of ram for the same stage. This runtime performance requirement would be prohibitive for more complicated model organisms such as zebrafish, which contains tens of thousands of cells (Keller *et al.* 2008). Extensive profiling and runtime simulations suggest, however, that the majority of this performance requirement comes from the overhead of accessing the memory of the observed and featured transformed image from the bottom up components. Recent work has shown that the increased memory bandwidth speeds and massive parallel capabilities of graphical processing units would be a more practical solution for these methods (Suchard *et al.* 2010). To explore this possibility, these methods were rewritten in OpenCL and run separately— resulting in a 25- to 100-fold increase in throughput when executed on the graphical processing unit. These results are encouraging and suggest that when combined with other modifications (*e.g.*, finely tuned proposal mechanisms), such improvements would be capable of addressing the increased computational demands for more complicated organisms.

## Supplementary Material

Supporting Information
