# Supplementary material for: A High-Fidelity Cell Lineage Tracing Method for Obtaining Systematic Spatiotemporal Gene Expression Patterns in Caenorhabditis elegans
Source: G3 (Bethesda). 2013 May 1;3(5):851–63. doi: 10.1534/g3.113.005918 (PMC3656732; doi:10.1534/g3.113.005918)
Supplement: Supporting Information [file supp_3_5_851__index.html]

A High-Fidelity Cell Lineage Tracing Method for Obtaining Systematic Spatiotemporal Gene Expression Patterns in Caenorhabditis elegans — Supporting Information 

# A High-Fidelity Cell Lineage Tracing Method for Obtaining Systematic Spatiotemporal Gene Expression Patterns in *Caenorhabditis elegans*

## Supporting Information for Mace *et al.*, 2013

**Files in this Data Supplement:**

- Supporting Information - File S1 and Figure S1 (PDF, 251 KB)
- File S1 - Supporting methods (PDF, 115 KB)
- Figure S1 - Time varying Linkage Parameters (PDF, 194 KB)
