# Supplementary material for: A High-Fidelity Cell Lineage Tracing Method for Obtaining Systematic Spatiotemporal Gene Expression Patterns in Caenorhabditis elegans
Source: G3 (Bethesda). 2013 May 1;3(5):851–63. doi: 10.1534/g3.113.005918 (PMC3656732; doi:10.1534/g3.113.005918)
Supplement: Supporting Information [file supp_g3.113.005918_005918SI.pdf]

G3: Genes | Genomes | Genetics

A High Fidelity Cell Lineage Tracing Method for Obtaining Systematic Spatiotemporal Gene Expression Patterns in *Caenorhabditis elegans*

Daniel L Mace, Peter Weisdepp, Louis Gevirtzman, Thomas Boyle and Robert H Waterston

Department of Genome Sciences, University of Washington, Seattle, WA

DOI: 10.1534/g3.113.005918

File S1

Supplemental Methods

Notation

Throughout this manuscript, we use various superscripts to denote group elements.

$$\begin{aligned} |g| &= \textit{generative component} \\ |d| &= \textit{discriminative component} \\ |l| &= \textit{linking component} \\ |s| &= \textit{size, spatial interaction, and cell number component} \\ |e| &= \textit{time to event component} \end{aligned}$$

In several cases, the superquadric kernel function $f(l(c_j),x)$ is used to describe a weighted value based on the normalized distance from the center of each nucleus where $l(\cdot)$ is the 3D location index and x is a location.

$$f(l(c_j),x) = \begin{cases} 0 & \\ 1 & S_e^j(c_j,x_i) > \tau_0 \\ exp\left\{-0.5\frac{\left(S_e^j(c_j,x_i)-\tau_0\right)}{\left(\tau_1-S_e^j(x_j)\right)}\right\} & S_e^j(c_j,x_i) < \tau_1 \\ & otherwise \end{cases}$$

$\tau_{\cdot}$ are arbitrary constants. S_e^j is an anisotropic superquadric normalized distance function from nucleus c_j to some arbitrary point v

$$S_c^j(v) = \left(\left(\left(\frac{v^x}{c_j^{a_1}}\right)^{c_j^{2e_2}} + \left(\frac{v^y}{c_j^{a_2}}\right)^{c_j^{2e_2}}\right)^{\frac{c_j^{e_2}}{c_j^{e_1}}} + \left(\frac{((v^z+p_0)*p_1)}{c_j^{2e_1}}\right)\right)^{c_j^{e_1}/2}$$

This distance function is a direct extension of the normalized superquadric distance function Jaklic et al. (2000) where the z distance value is modified by ρ_0 and ρ_1 , to account for larger z attenuation effects. v is the affine transformed point that is projected into the superquadrics coordinate space: $v = A'_{c_j}x - l(c_j)$, where A'_{c_j} is the inverse rotation matrix of c_j . A more generalized version of this distance function is used for some components:

$$\Psi\left(l(c_j), l(\mu_i^o), \lambda_.\right) = \begin{cases} \lambda_0 & if\ D_e(c_j, x_i) > \tau_0^o \\ \lambda_1 & if\ D_e(c_j, x_i) < \tau_1^o \\ \lambda_2 * \exp\left\{-0.5 \frac{D_e(c_j, x_i) - \tau_0}{\tau_1 - D_e(c_j, x_i)}\right\} & otherwise \end{cases}$$

$\lambda_0 = 0, \lambda_1 = 1, \lambda_2 = 1$ if not specified otherwise. D_e is the euclidean distance function.

Full Model

This section details the full specification of the model, including all parameters and hyperparameters that were used for training the model. The full conditional posteriors were obtained using the standard Markov blanket, and a slice sampler wrapped around a simulated annealing method was used to fit the parameters for each individual component.

Bottom Up Generative Model

$$\begin{aligned}
\mu_i^{|g|} &= \mu_b^{|g|} + \textstyle\sum_j^{m_t} \gamma\left(c_j, l(\mu_i^g) \middle| \tau_.^{|g|}\right) c_j^\mu \\
\tau^{|g|} o_. &\sim N\left(\mu_{\prime,1}^{|g|}, \sigma_{\prime,1}^{2,|g|\cdot}\right) \\
c_j^\mu &\sim N\left(\mu_{\prime,2}^{|g|}, \sigma_{\prime,2}^{2,|g|}\right) \\
\sigma_{\prime,1}^{2,|g|} &\sim IG\left(\alpha_1^{|g|}, \beta_1^{|g|}\right) \\
\mu_b^{|g|} &\sim N\left(\mu_{\prime,3}^{|g|}, \sigma_{\prime,3}^{2,|g|}\right) \\
\sigma_b^{2,|g|} &\sim IG\left(\alpha_2^{|g|} \beta_2^{|g|}\right) \\
p\left(x_i \middle| \mu^{|g|}\right) &\propto \mathcal{L}_1\left(\mu_1^{|g|}, b_1\right) \mathcal{L}_2\left(\mu_2^{|g|}, b_2\right) \\
L_1\left(x, \mu^{|g|}, b_1\right) &\propto \exp\left(-\psi\left(x, \mu^{|g|}, b_1, \pi\right)\right) \\
L_2\left(\hat{x} \middle| \widehat{\mu^{|g|}}, b_2\right) &\propto \exp\left(-\psi\left(x, \widehat{\mu^{|g|}}, b_2, \pi\right)\right) \\
\psi\left(x_i, \mu_i^{|g|}, b_., \pi\right) &= \left(\pi_i^1 \left|x_i - \mu_i^{|g|}\right| / b_. + (1 - \pi_i^1) \left|x_i - \mu_b^{|g|}\right| / b_.\right) \\
\widehat{\mu_i^{|g|}} &= \Delta\left(\mu_i^{|g|}\right) \\
\pi &= \textstyle\sum_j^{m_t} \left[f\left(c_j, l\left(\mu_i^{|g|}\right)\right) 1/V(c_j)\right] \\
\sqrt{b} &\sim \mathcal{IG}(\alpha_3^{|g|}, \beta_3^{|g|})
\end{aligned}$$

Discriminative Model

$$\begin{aligned}
\hat{r_i} &= \frac{(r_i - r_0)}{a_0} \\
R_. &\sim N\left(\mu^{|d|}, \Sigma^{|d|}\right)
\end{aligned}$$

$$\mu^{|d|} = 1 - \beta_1^{|d|} S_e(r)$$
$$\Sigma_{i,j}^{|d|} = \sigma^{2,|d|} k_1^{|d|}(i,j) k_2^{|d|}(i,j) k_3^{|d|}(i,j) + \epsilon^{|d|}$$
$$k_1^{|d|}(i,j) = 1 - \gamma_0^{|d|} \left| z(\hat{r_i}) - z(\hat{r_j}) \right|$$
$$k_2^{|d|}(i,j) = exp\left\{ -\frac{D_a(i,j)^2}{2\sigma_1^{2,|d|}} \right\}$$
$$k_3^{|d|}(i,j) = \exp\left\{ -\frac{\left(S_e(r_i) - S_e(r_j) \right)^2}{2\sigma_2^{2,|d|}} \right\}$$
$$y^{|d|} = \omega_0^{|d|} + \omega_1^{|d|} \log\left(p(\hat{r}.) \right) + \omega_2^{|d|} t(r_0)$$
$$t(r_0) = exp\left\{ \frac{\left(\tau_0^{|d|} - r_0 \right)}{\tau_1^{|d|}} \right\}$$
$$p^{|d|}(c \mid \cdot) = 1/\left(1 + \exp(-y_j) \right)$$
$$\sigma^{2,|d|} \sim IG\left(\alpha_0^{|d|}, \beta_0^{|d|} \right)$$
$$\epsilon^{|d|} \sim IG\left(\alpha_0^{|d|}, \beta_0^{|d|} \right)$$
$$\tau_.^{|d|} \sim N\left(\widehat{\mu_1^{|d|}}, \widehat{\sigma_1^{2,|d|}} \right)$$
$$\omega_.^{|d|} \sim N\left(\widehat{\mu_2^{|d|}}, \widehat{\sigma_2^{2,|d|}} \right)$$

$$A = \begin{bmatrix} cos(\theta_0)cos(\theta_1) & sin(\theta_0) & cos(\theta_0)sin(\theta_1) \\ sin(\theta_0)cos(\theta_1) & cos(\theta_0) & sin(\theta_0)sin(\theta_1) \\ sin(\theta_1) & 0 & cos(\theta_0) \end{bmatrix}$$

$$\theta_0 = \{0, \ldots, 2\pi\}, \theta_1 = \{0, \ldots, \pi\}$$

and D_a is the angular distance function.

Top Down Model

Time Varying Parameters All the time varying parameters described here use a series of k equally spaced knot points in conjunction with a Gaussian kernel function. Every time varying parameter follows the same convention and parameter/hyper parameter configuration described below

$$\theta_t = \sum_i^k w_{i,t} \phi_i$$
$$w_{i,t} = \frac{k(i,t)}{\sum_j^{m_t} k(j,t)}$$

$$k(i,j) = exp^{[-0.5D_e(i,j)/\sigma^{2,r}]}$$
$$\Phi_i - \Phi_{i+1} \sim N(\mu_0^r, \sigma_0^{2,r})$$
$$\Phi \sim N(\mu_1^r, \sigma_1^{2,r})$$

Number of Nuclei Model

$$M_t \sim N(\mu_{t,3}^{|s|}, \sigma_{t,3}^{2,|s|})$$

Nucleus Size Model

$$C_1^a \sim N(\mu_{t,1}^{|s|}, \sigma_{t,1}^{2,|s|})$$

Nucleus Interaction Model

$$p(c|\lambda_1,\cdot) = \frac{exp\{\sum_{i,j} h(c_i, c_j)\}}{Z}$$
$$h(c_{j,t}, c_{k,t}) = \Psi(c_{j,t}, c_{k,t}, 0, \infty, \lambda_s)$$

Time to Event Model

The training of the time to event component differs slightly from the other components in that many of the observations are not directly observed. On both the start of the image series (time 1) and the end of the image series at time n , the start and the end of the leafs of the lineage are not observed. To account for this, each observation is split into multiple equally weighted observations, and the type and timing of the event are imputed. During the imputation, the timing of the event is sampled from a truncated normal based on the last observed time. To adjust for the strong bias of the AB lineage in cell numbers and synchrony of division times, pseudo counts for each observed event in the C, D, MS and E lineages were added to provide a more realistic representation of the observed division times. In addition, pseudo-count observations are added to account for polar body cells that are not present in the trained data. Polar body cells are a natural by-product of early oogenesis and are much smaller sized cells that eventually die off. Depending on the quality and type of reporter construct, these cells appear in different numbers and persist for a varying amount of time (some persisting throughout embryonic development). 8 pseudo counts are added to account for this variability, 4 cells with imputed death types, and 4 cells that are at final differentiation.

$$p(B_i|e,\cdot) = \pi_{S(B_i)}^{|e|} p_{S(B_i)}^{|e|}(d(B_i))$$
$$\pi_i^{|e|}(i) = \frac{\exp\{\sum_l^m k_{i,l}^{|e|}(t)\}}{\exp\{\sum_j^3 \sum_l^m k_{i,l}^{|e|}(t)\}}$$
$$p^{|e|}(c_j|\,\cdot) \propto \exp\left\{-\frac{(o(c_j) - \mu^{|e|})^2}{\sigma^{2,|e|}}\right\}$$

$$\mu^{|e|} = \lambda_0^{|e|} + \sum_i^m k_i^{|e|}\left(d(c_j)\right)\lambda_1^{|e|}$$

$$\sigma^{2,|e|} = exp\left\{\gamma_0^{|e|} + \sum_i^m k_l^{|e|}(d(c_j))\gamma_l^{|e|}\right\}$$

During the decoding process, the base probability is changed to a mixture of the trained time to event model and base probability:

$$p(c|e,\cdot) = \lambda^0 + \lambda^1 \pi_v^e p_v^e(S(B_i))$$

This mixture provides a minimum allowed probability for the time to event model λ_0 , and prevents the time to event component of the model from over-correcting when encountering false positives and false negatives. Linkage Model

$$p(c_t|c_{t-1},\cdot) = \exp\left(\psi(x)\right)$$

$$\psi(x) = \sum_k^n \left(g_k^{|l|}\beta_k^{|l|}h_n^{|l|}(\cdot)\right)$$

$$h_k^{|l|} = -\left(x_k^{|l|} - \mu_k^{|l|}\right)^2 / \sigma_k^{2,|l|}$$

$$\mu_k^{|l|} = \lambda_{1,k}^{|l|} + \lambda_{2,k,t}^{|l|} + \lambda_{3,k,t}^{|l|} e_{t,t-1}$$

$$\sigma_k^{2,|l|} = \exp\left\{\gamma_{1,k}^{|l|} + \gamma_{2,k,t}^{|l|} + \gamma_{3,k,t}^{|l|} e_{t,t-1}\right\}$$

$$\beta^{|l|} \sim N\left(\mu_1^{|l|}, \sigma_1^{2,|l|}\right)$$

$$\lambda_{,k}^{|l|} \sim N\left(\mu_2^{|l|}, \sigma_2^{2,|l|}\right)$$

$$\gamma_{,k}^{|l|} \sim N(\mu_3^{|l|}, \sigma_3^{2,|l|})$$

The full time varying trained parameters for the model are shown in Supplemental Figure 2.

The covariability function, $g_k^{|l|}$, helps correct for interactions between individual features. While these interactions are often minor and have minimal impact on the training of the model, failing to account for these interactions can result in odd shaped divisions called during the decoding process (often due to false-positives/false negatives that the model does not correct for appropriately. The interaction between variables is modelled as:

$$g_k^{|l|} = \upsilon_1^{|l|} + \upsilon_2^{|l|} exp\left(\sum_j^n \phi_j^{|l|}\beta_j^{|l|}h_j^{|l|}(\cdot)\right)$$

where ϕ_j is a binary indicator value for the terms. Four interaction terms are modelled: division angle and difference of mother/daughter cells; division angle and distance of mother/daughter cells, distance of mother/daughter cells and difference of distance of mother/daughter cells, and distance of mother/daughter cells and the difference in the radius of the cells.

Initial Seeding

Before the decoding of the algorithm enters its main iterative phase, an initial seeding step is run. This initial seeding proposal is run for two reasons: to run a fast proposal for individual nuclei that is independent of time; and to obtain an initial seeding of the nuclei to fit a model for the normalized offset and rate of the image series. For each time point t, let $\breve{c}_{\cdot,t}$ be the proposed points. The proposal seeding is done using the proportional probability of the discriminative model, nuclei size, and the spatial interaction: $p(\breve{c}_{\cdot,t}|,\cdot) \propto p(\breve{c}_{\cdot,t}|d,\cdot)p(\breve{c}_{\cdot,t}|\lambda,\cdot)p(\breve{c}_{\cdot,t}|s,\cdot)$. An iterative algorithm is run that proposes to add a new point $c_{j,t}$ to the seed data, $\breve{c}_{\cdot,t}{}' = \breve{c}_{\cdot,t} \cup c_{j,t}$, where the new point is acceptance ratio is above a certain cutoff ρ_c . The algorithm terminates for time t when a new point has not been accepted in the last k attempts.

Empirical Proposal Distributions While all moves are reversible, a number of moves break detailed balance by using empirical proposal distribution to bias the move towards more probable regions. These moves are necessary to bring the computational demands of the decoding down to reasonable levels.

Decoding

Reversible Jump Markov Chain Monte Carlo Methods (RJMCMC) are used to decode the lineage from any given trace. RJMCMC methods differ from traditional MCMC methods in that special consideration must be made to properly normalize the trans-dimensional jumps in the model. In many cases with Bayesian methods, the normalizing constant can be dealt with by integrated out the prior information. In our particular case, this is not possible; and as a result, we use a composite space and pseudo-priors to allow for equivalent normalizing factors Godsill (2001). Let $p(\widehat{c'}|d,s,e)$ be a draw from the joint prior of the augmented data, and let

$$Z = \frac{1}{p(\widehat{c'}|d,s,e)}$$

be the normalizing ratio constant of a reversible jump move (in this example, the addition of a new point $j+1$ at time t to the model where $\widehat{c'} = \hat{c} \cup c_{j+1,t}$. The acceptance ratio of the move is then:

$$\alpha\left(\hat{c}, \widehat{c'}\right) = min\{1, \frac{p(x|\widehat{c'},g,\cdot)p(\widehat{c'}|d,\cdot)p(\widehat{c'}|s,\cdot)p(\widehat{c'}|l,\cdot)p(\widehat{c'}|e,\cdot)}{p(x|\hat{c},g,\cdot)p(\hat{c}|d,\cdot)p(\hat{c}|s,\cdot)p(\hat{c}|l,\cdot)p(\hat{c}|e,\cdot)} * Z\}$$

Z provides the missing normalizing constant for the distributions that result from the addition of the point that is missing in ĉ but present in $\widehat{c'}$ (e.g., the Gaussian prior on the major axis). For performance reasons, we simulate the most probable draw from the joint prior. While

this limits our ability to address uncertainty in the choice of model, the effect is mitigated by the fact that we are using a hybrid simulated annealing approach to sample the most probable configuration and are not directly concerned with the uncertainty of the lineage. Pure methods for properly calculating this constant using full detailed balance RJMCMC methods or bridge/path sampling are possible, but are not practical from a computational/memory perspective.

Sequential RJMCMC Moves

Both the add point and remove point use empirical proposals. Split, merge, and updating the points within a branch are done uniformly. The add point proposal proposes a region to add a new point. Let $S_{t,\cdot}$ be the set of all possible regions. The probability of a point being added at time t to region i is:

$$p^a(S_{t,i}|\cdot) \propto \pi_1 + (1-\pi_1)(1 - exp\left\{\phi_0^a \sum_j^{|S_{t,i}|} \epsilon_l^{|g|}(S_{t,i}, j) + \phi_1^a \sum_j^{|S_{t,i}|} \sum_k^m {}_{t+1}\kappa(k,j) log(p_{tte+1}(c'|c))\right\}$$

where $\epsilon^o_{l(j,S_{t,k})}$ is the residual from the generative observed image at the jth sub-location in region $S_{t,k}$, and p_{tte+1} is the proposed time to event ratio probability of adding a new hypothetical point and attaching it to the kth parent of time $t-1$:

$$p_{tte+1}(c'|c) \propto \pi_2 + (1-\pi_2)(1 - p(c|\cdot) \,/\, p(c'|\cdot))$$

where $\kappa(k,j)$ is a Gaussian kernel function describing the weight from the center of the kth parent to location j and $\phi_\cdot^a < 0$ are negative constants that invert the log probabilities. A new point is then drawn uniformly from this region.

The proposal to remove a nuclei is based on the current state of the discriminative model. The probability to propose removing nuclei j at time t is:

$$p^r(c_{j,t}|\cdot) = exp\{\phi_0^a log(\pi_3 + (1-\pi_3)p(c_{j,t}|d,\cdot))\}$$

where $\phi_0^r < 0$.

The split move splits a single nuclei into two nuclei. The location of the two new nuclei is set to be orthogonal to the parent point. Let $W \sim U(2,5) * c_{i,t}^{a_1}$ be a magnitude that is drawn from a uniform distribution times the major axis length of the previous point, and R be a unit vector drawn from uniformly from the unit sphere. The locations for the new nuclei j and k are then a function of previous location, the magnitude, and the unit vector $c_{j,t}^{x,y,z} = c_{i,t}^{x,y,z} + R * w$ and $c_{k,t}^{x,y,z} = c_{i,t}^{x,y,z} - R * w$.

Non-sequential RJMCMC Moves

Empirical proposals for selecting a new point are used for: adding a new branch, adjusting the beginning of a branch, and adjusting the end of a branch. Proposing a branch to update or delete are selected uniformly from all possibly branches. The proposal distribution for selecting a branch $\widehat{c_j}$ to update is:

$$p_{be}(\widehat{c_\iota}) \propto \pi_4 + (1-\pi_4)\left(1 - exp\{\phi_0^{be} p\left(b_p\left(\widehat{c_j}\right)\middle|l\right) + \phi_1^{be} p\left(\widehat{c_j}\middle|e\right)\}\right)$$

where b_p is the beginning parent (the mother cell of a division). These proposals are used for relinking both the beginning and end of the branch. For adding a new branch, an empirical proposal is used for selecting the initial seed point based on a set of i past sequential rejected points at time : $R_{i,t}$. During the chronological stage, when rejecting a move from ĉ to ĉ′ in the add stage, the point $c_{i,t}$ has a probability of adding to the rejected point list. This acceptance probability of adding a point to the set:

$$\alpha_j(R_{t,i}, R'_{t,i}) = \frac{p(x,\mu|\hat{c}')p(\hat{c}'|d)}{p(x,\mu|\hat{c})p(\hat{c}|d)}$$

This proposal biases to points that have strong evidence from the image of existing but are often rejected due to topological constraints (linkage, time to event, etc.).

While the length of a new branch λ_1 , as well as the distance to look for relinking the start and end of each branch λ_2 , are drawn from a modified Poisson distribution, each move has an empirically probability of extending the length of this branch based on the following: Let $\widehat{c'}$ be the proposed new configuration from a new branch, or the start/end relinking moves, where ĉ is the original configuration. We extend the addition of a new branch, or the relinking of the start end by $\lambda_.$ with probability , where b_r is the probability ratio of the bottom up components:

$$b_r = \frac{p(x|\widehat{c'},g\cdot)p(\widehat{c'}|d,\cdot)}{p(x|\hat{c},g\cdot)p(\hat{c}|d,\cdot)}$$

and t_p is the product of the link probability and time to event of the proposed new branch/relinking step. This empirical proposal extends regions where strong evidence from the data that a point should exist, but whose linkage and time to event components suggests the incorrect parent (and hence, that it should be in a different time point).

Relinking Empirical Moves

During the relinking phase, a point can be added whose true parent is bound incorrectly to a child, or whose parent will unlikely be accepted due to a previous miscalled division. To overcome these issues, we've designed several empirical proposals that will allow an already

bound child to choose a new parent. Let $E_p(c_{j,t}), E_{c_0}(c_{i,t}), E_{c_1}(c_{j,t})$ be the current parent, first, and second (if exists) child of a cell nucleus and $|E_c|$ is the number of children. When proposing a new parent, $\widehat{E_p}(c_{j,t})$, each child of the current parent $c_{j,k} = E_c(\widehat{E_p}(c_{j,t}))$ has a probability of entering its own relinking phase of:

$$p_{r(c)}\left(E_c(\widehat{E_p}(c_{j,t}))\right) = exp\left\{\phi_0^{r(c)} \log\left(\pi_0^{r(c)}\lambda_0^{r(c)}\gamma_0^{r(c)} + \pi_1^{r(c)}\lambda_1^{r(c)}\gamma_1^{r(c)}\right) + \log\left(\lambda_2^{r(c)}\gamma_2^{r(c)}\right)\right\}$$

where $\lambda_\cdot^{r(c)}$ are constant weight factors, and $\gamma_0^{r(c)}, \gamma_1^{r(c)}$ are the link and time to event probability improvement ratios:

$$\gamma_0^{r(c)} = \frac{p(c_{i,t}|E_p(c_{i,t}),l)^2}{p(c_{i,t}|E_p(c_{i,t}),l)p(c_{j,t}|E_p(c_{j,t}),l)}$$
$$\gamma_1^{r(c)} = \frac{p(\hat{c}'|e)}{p(\hat{c}|e)}$$

When proposing a new parent, it is possible that the probability of the link is good, but the time to event model leads to a decreased probability of the true parent being selected. In many situations, this occurs when a parent/child relationship in a previous or future time point is incorrect. This is also addressed through empirical proposals. When a new proposed parent increases the number of children (a continuation to a division), a proposal to relink a previous or future cell within the branch is made:

$$p_{r(d)} = \exp\left\{\phi_0^{r(d)} log\left(\pi_0^{r(d)}\lambda_0^{r(d)}\gamma_0^{r(d)} + \pi_1^{r(d)}\lambda_1^{r(d)}\gamma_1^{r(d)}\right) + \phi_1^{r(d)} log\left(\lambda_2^{r(d)}\gamma_2^{r(d)}\right) + \phi_2^{r(d)} log\left(\lambda_3^{r(d)}\rho_0^{r(d)}\right) + \phi_3^{r(d)} log\left(\lambda_4^{r(d)}\tau_0^{r(d)}\right)\right\}$$

$\gamma^{r(d)}$ are the improvements of the linkage as described above, $\rho_0^{r(d)}$ is the ratio of the previous division to the geometric mean of the divisions before and after(if it exists) and $\tau_0^{r(d)}$ is a log weighted probability based on the improvement of the link and the change in the time to event distributions:

$$\rho_0^{r(d)} = \frac{p(c_{j,t}|\widehat{E_p}(c_{j,t}),l)^2}{p\left(E_p\left(N\left(I_b(c_{j,t})\right)\right)\middle|\widehat{E_p}(c_{j,t}),l\right)p\left(V\left(I_b(c_{j,t})\right)\middle|\widehat{E_p}(c_{j,t}),l\right)}$$
$$\tau_0^{r(d)} = \gamma_1^{r(d)}\frac{1}{1+exp\left(log\left(\gamma_0^{r(d)}\right)\right)}$$

$\tau_0^{r(d)}$ increases the probability of a past future relink if the link probability has improved, but the time to event probability has gotten worse. This ensures that a previous time relinking will only be attempted if the transition to a division is restrictive due to a division that recently occurred.

To maintain balance, we must also allow the reverse: when a new parent is proposed, and the old parent transitions from 2 children to 1 (division to a continuation), a relinking step that allows a previous or future division $r(a)$ occurs with probability:

$$p_{r(a)} = \exp\left\{\phi_0^{r(a)}\, log\left(\pi_0^{r(a)}\lambda_0^{r(a)}\gamma_0^{r(a)} + \pi_1^{r(a)}\lambda_1^{r(a)}\gamma_1^{r(a)}\right) + \phi_1^{r(a)}\, log\left(\lambda_2^{r(a)}\gamma_2^{r(a)}\right) + \phi_2^{r(a)}\, log\left(\lambda_3^{r(a)}\tau_0^{r(a)}\right)\right\}$$

$\tau_0^{r(a)}$ is a slightly modified version of removing a division. Instead of a straight value for the time to event probability, a slightly modified value is used to allow for an increased probability of adding a past division when it nears the expected division time:

$$\tau_0^{r(a)} = \gamma_1^{r(a)} \frac{1}{1 + \exp\left\{log\left(\gamma_0^{r(a)} - \beta_0^{r(a)}\right)\right\}}$$

Decoding algorithm

The decoding algorithm is based on single time and multi time reversible jump Markov chain Monte Carlo methods. The specifics of the algorithm are described below using pseudo code and briefly described here. The program initiates with a proposal step (PROPOSAL) that burns in a series of proposal points using the discriminative distribution across all the points. This is followed by a fit of the normalized time to a linear model (NORMALIZED_FIT). The method then enters an iterative procedure, where for every time point, for a variable number of iterations per time point, sequential and non-sequential moves are sampled with fixed probability constants (SAMPLE_FULL). After running through all time points, the algorithm enters a final iteration where only non-sequential moves are sampled (SAMPLE_NON).

```
PROPOSAL()
NORMALIZED_FIT()
for i ← 0 to k do
        for j ← 0 to β_0 + β_1 * μ_t^{|q|} do
                SAMPLE_FULL()
        if i mod 10 = 0 then
                NORMALIZED_FIT()
NORMALIZED_FIT()
for i ← 0 to n do
        SAMPLE_NON()
```

Automated error analysis

To assess performance of individual methods, we developed an automated method for calculating the error rate. This method calculates the error rate by comparing the lineage tree of each method (SALT, WS-SN,BS-SN) to the manually edited ”ground truth” lineage. The ground truth images were created by manual curation of an automated lineage using semi-automated correction tools. In accessing the performance, we decompose the error rate into two types of errors: detection errors (false positive, false negative), and relinking errors. The error rates are calculated by determining an identifiable mapping between the ground truth and the automated method. Let $c_{j,t}^{m}$ be the jth point at time t in the automated method, and let $c_{k,t}^{g}$ be the kth point at time t in the ground truth data set. Points $c_{j,t}^{m}, c_{k,t}^{g}$ are identifiable, $I_a(c_{j,t}^{m}, c_{k,t}^{g})$, if the euclidean distance between them is smaller than the maximum major axis $I(c_{j,t}^{m}, c_{k,t}^{a}) \Leftrightarrow dist(c_{j,t}^{a}, c_{k,t}^{g}) < max(c_{j,t}^{m,a_1}, c_{k,t}^{g,a_1})$. Let I be the set of points from the automated method that have an identifiable ground truth point, where $i(c_{j,t}^{[m,g]})$ is a function that returns the respective identifiable cell pair or null if no such identification exists. A precursor false positive error exists if a nucleus in the automated method does not have a respective identifiable nucleus in the ground truth that occurs for more than 2 time points $c_{j,t}^{m} \in \widehat{FP} | i(c_{j,t}^{m}) = \emptyset, i(e_p(c_{j,t}^{m})) = \emptyset$. Conversely, a precursor false negative error exists if a cell exists in the ground truth but has no identifiable cell in the automated method $c_{j,t}^{g} \in \widehat{FN} | i(c_{j,k}^{g}) = \emptyset, i(e_p(c_{j,k}^{g})) = \emptyset$. The full false positive and false negatives are then aggregated into false positive and false negative sequential sets $(FP, FN$). Let FN' be the set of all false negatives that start a sequential unit, $p \in FN'$ if $\in FN, e_p(p) \ni \widehat{FN}$. We let $f(p)$ be the set of all sequential elements such that $(p) = \big(p, e_c(p), \dots, e_c(\cdot)\big) \in \widehat{FN}$. The full false positive and false negative aggregate sets are all sequential sets of the precursor false negative sets: $(p) \in FN | \forall p \in FN'$. A similar composition occurs for the false negatives, with the exception that we only count false positives that end in a cell death: $c_{j,t}^{m} \in FP \Leftrightarrow |V_{c_{j,t}^{m}}| = 0$. False positives that do not result in a death but are eventually moved back to an original cell are referred to as move-jump link errors and are counted as described below. We split linking errors into four categories: division errors, rebirth-division errors, move-jump errors, and all other linking errors. Division errors occur when the expected daughter is linked to a previous cell: $e_p(i(c_{j,t}^{m})) \ni e_p(c_{j,t}^{m})$ if $|e_c(e_p(i(c_{j,t}^{m})))| = 2$. These mark errors where a daughter of a division is assigned to the wrong parent. We further denote link-birth errors as occasions where the division is missed (a false negative), and one or both of the daughters are linked to a new cell: $I_b(e_p(i(c_{j,t}^{m}))) \neq I_b(e_p(c_{j,t}^{m}))$ and $(e_p(i(c_{j,t}^{m}))) \in FN$. Move jump errors occur when a cell jumps out of location for a short time before moving back to it’s original location: $e_p(I_b(c_{j,t}^{m})) \in FP$. For all remaining linking errors, we consider a link error to be an identifiable cell whose parent cell identity does not match up in the automated methods and ground truth and whose actually parent is not the branch parent of the

identifiable cell: $I_b(e_p(c_{j,t}^m)) \neq I_b(e_p(i(c_{j,t}^m)))$ and $I_b(e_p(c_{j,t}^m)) \ni I_b(e_c(V_{e_p(e_p(i(c_{j,t}^m)))}))$. The prerequisite on having 2 subsequent cells being missing for a detection error and allowing for parent identifiable cells to belong to previous branches is to allow flexibility in division calls: cells undergoing division often appear to divide within a range of 2 time points and are often so closely spaced that the ”ground truth” could occur in either time point (this is especially the case in z-axis divisions, where the increased z attenuation often makes two daughter cells appear to be a larger mother cell.)

Imaging setup and strain information

Images are captured as described in (Murray et al., 2008). Full information on strains including detailed information on the underlying constructs can be obtained at: http://epic2.gs.washington.edu/Epic2/. Late edited series can be obtained at: http://epic2.gs.washington.edu/Epic2/?SeriesList=MaceList

Tables

Method	245 Sulston Time		330 Sulston Time	
	Detection	Linking	Detection	Linking
SALT	6.8	10.8	49.6	119.0
BS-SN	7.3	52.8	106.8	536.7
WS-SN	93.3	132.1	652.8	1101.0

Table 1: Evaluation Accuracy: Table output of the graph shown in Figure 5

Method	245 Sulston Time				330 Sulston Time			
	DE	RDE	MJE	LE	DE	RDE	MJE	LE
SALT	4.1	0.4	2.8	3.4	19.6	3.3	35.1	61.1
BS-SN	17.2	7.1	4.7	23.8	63.8	18.6	71.3	383.0
WS-SN	29.1	17.4	15.0	70.6	85.5	92.0	97.4	826.1

Table 2: Linking Accuracy: In depth analysis of the linking errors. DE=Division Error, RDE=Rebirth-Division Error, MJE=Move Jump Error, LE=Linking Error

References

Simon J. Godsill. On the Relationship Between Markov Chain Monte Carlo Methods for Model Uncertainty. *Journal of Computational and Graphical Statistics*, 10:230–248, 2001.

Ales Jaklic, Ales Leonardis, and Franc Solina. *Segmentation and recovery of superquadrics*.

Kluwer Academic Publishers, 2000. ISBN 9780792366010.

John I. Murray, Zhirong Bao, Thomas J. Boyle, Max E. Boeck, Barbara L. Mericle, Thomas J. Nicholas, Zhongying Zhao, Matthew J. Sandel, and Robert H. Waterston. Automated analysis of embryonic gene expression with cellular resolution in C. elegans. *Nature Methods*, 5(8):703–709, June 2008. ISSN 1548-7091. doi: 10.1038/nmeth.1228.

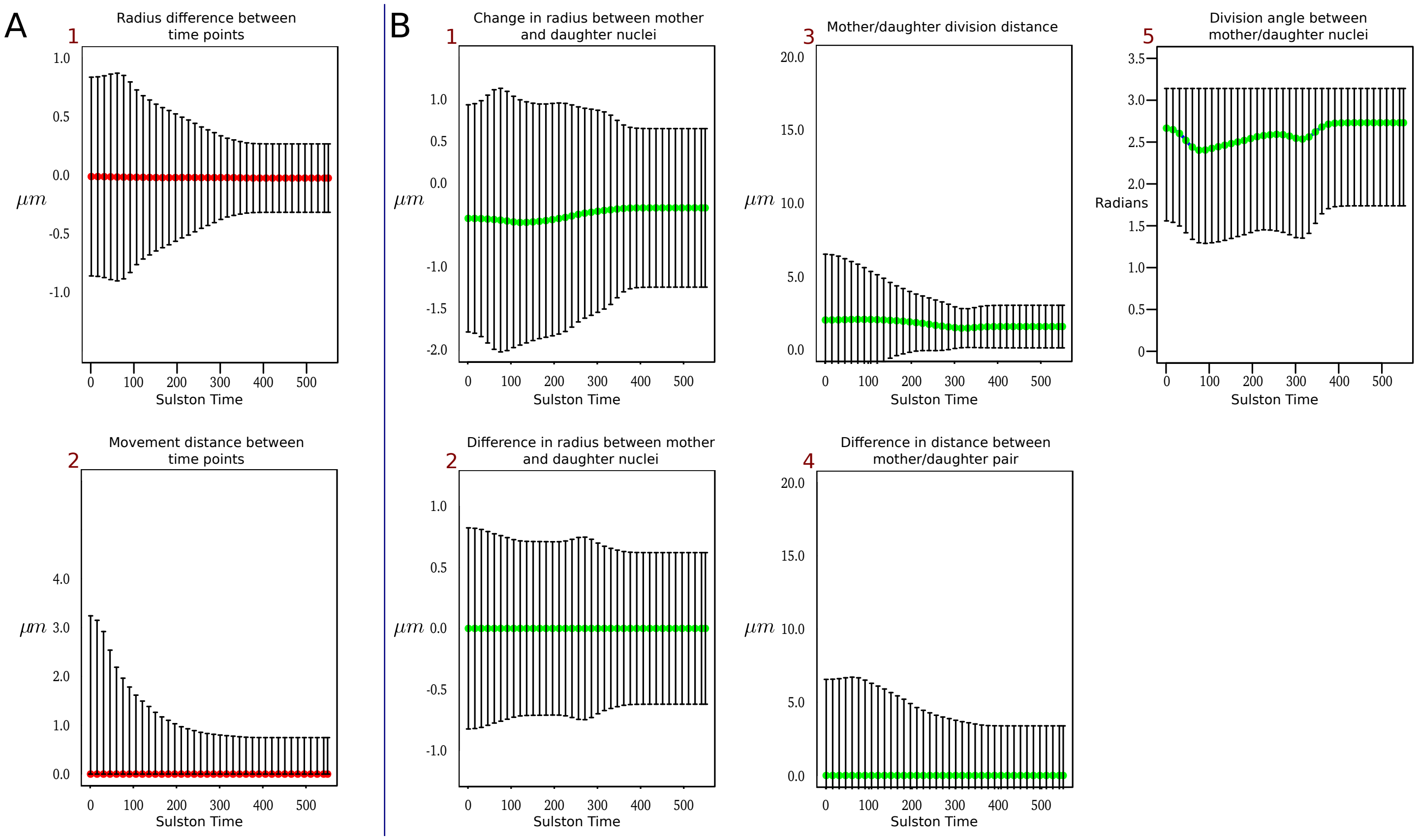

Figure S1 Time varying Linkage Parameters: Individual time varying parameters for the features used in the conditional random field (CRF) linkage distribution. The x axis is the normalized sulston time, while the y axis are the actual mean and one standard deviation values for the features (radians for the division axis, microns in all others). As the CRF is a discriminative model, the features represent the parameters needed to discriminate correct links through time from possible incorrect links. (A) The continuation linkage model consists of two features: (A.1) the change in radius of a nucleus between two time points, (A.2) the movement of each nucleus across time. (B) The division parameters. (B.1) Change in radius between mother and daughter nuclei, (B.2) Difference in change of radius between mother/daughter nuclei, (B.3) The distance each daughter cell moves from the mother nuclei (B.4) The difference in distance of the movement of the nuclei (B.5) The division axis between the mother and the two daughter nuclei. This division axis is the inner angle of the division with the mother nucleus as the center vertex as compared to the standard A/P rotation commonly used in the *C. elegans* community.
