# Supplementary material for: A High-Fidelity Cell Lineage Tracing Method for Obtaining Systematic Spatiotemporal Gene Expression Patterns in Caenorhabditis elegans
Source: G3 (Bethesda). 2013 May 1;3(5):851–63. doi: 10.1534/g3.113.005918 (PMC3656732; doi:10.1534/g3.113.005918)
Supplement: Supporting Information [file supp_g3.113.005918_FigureS1.pdf]

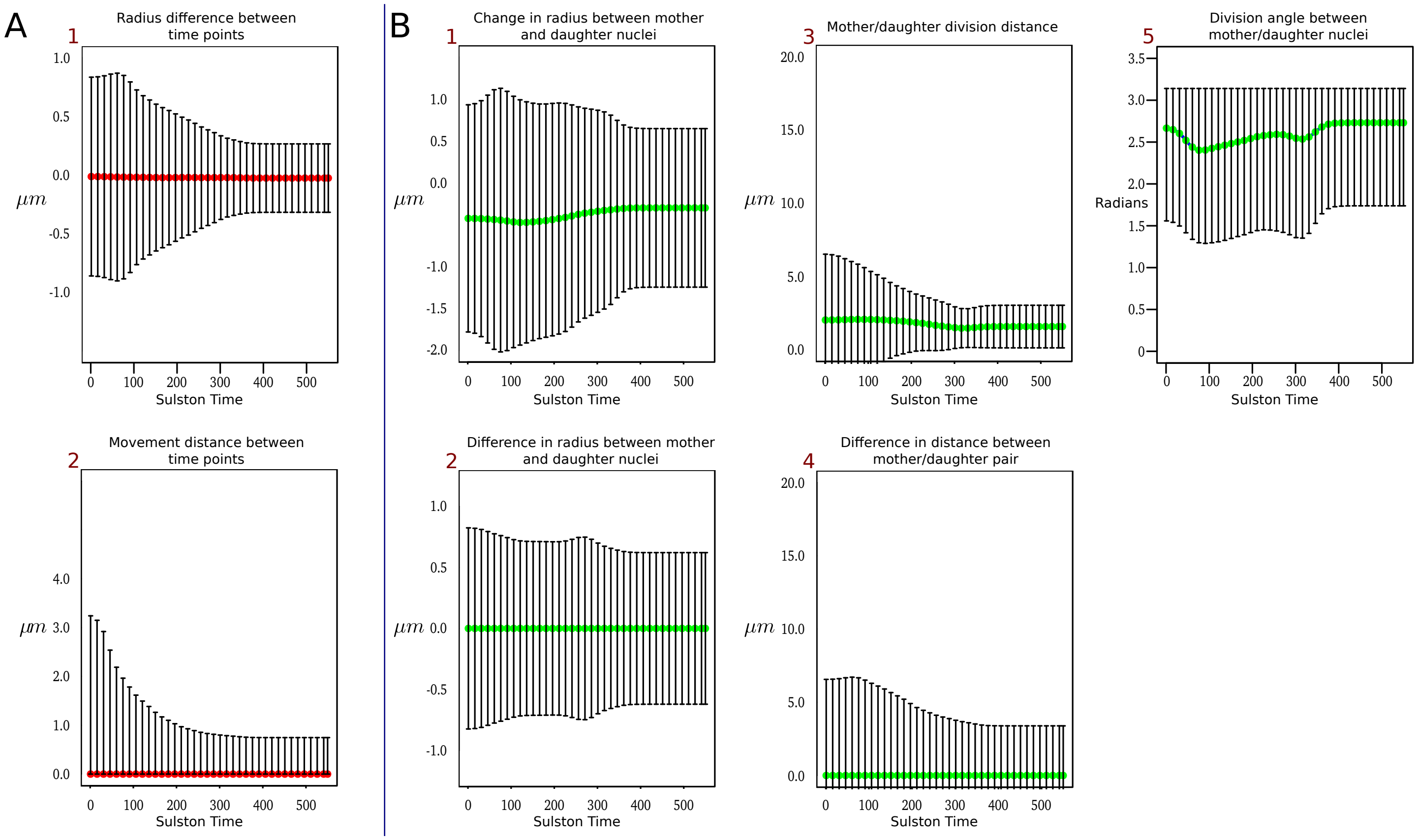

Figure S1 Time varying Linkage Parameters: Individual time varying parameters for the features used in the conditional random field (CRF) linkage distribution. The x axis is the normalized sulston time, while the y axis are the actual mean and one standard deviation values for the features (radians for the division axis, microns in all others). As the CRF is a discriminative model, the features represent the parameters needed to discriminate correct links through time from possible incorrect links. (A) The continuation linkage model consists of two features: (A.1) the change in radius of a nucleus between two time points, (A.2) the movement of each nucleus across time. (B) The division parameters. (B.1) Change in radius between mother and daughter nuclei, (B.2) Difference in change of radius between mother/daughter nuclei, (B.3) The distance each daughter cell moves from the mother nuclei (B.4) The difference in distance of the movement of the nuclei (B.5) The division axis between the mother and the two daughter nuclei. This division axis is the inner angle of the division with the mother nucleus as the center vertex as compared to the standard A/P rotation commonly used in the *C. elegans* community.
